# Exploring the provision and structure of paediatric critical care outreach teams (PCCOTs) in the UK and Ireland: a national questionnaire study

**DOI:** 10.1136/bmjpo-2025-003920

**Published:** 2025-12-21

**Authors:** Bethan Holmes, Julie Christine Menzies, Susan Neilson, Heather Duncan, Lucille M Kelsall-Knight

**Affiliations:** 1 Department of Nursing and Midwifery, University of Birmingham, Birmingham, UK; 2 Paediatric Critical Care Outreach Team (PCCOT), University Hospitals of Leicester NHS Trust, Leicester, UK; 3 Paediatric Intensive Care Unit, Bristol Royal Hospital for Children, Bristol, UK; 4 University of the West of England, Bristol, UK; 5 University of Birmingham, Birmingham, UK; 6 PICU, Birmingham Children’s Hospital NHS Foundation Trust, Birmingham, UK

**Keywords:** Child, Cross-Sectional Studies, Nursing, Child Health, Health services research

## Abstract

**Background:**

Failure to recognise and respond to early signs of critical illness contributes to preventable deaths in the UK, particularly among medically complex children. Critical care outreach teams (CCOTs) are multidisciplinary teams that manage deteriorating patients and support early care escalation. While well-established in adult services, Paediatric CCOTs (PCCOTs) remain under-researched. This study presents the first national evaluation of PCCOT provision and characteristics across tertiary paediatric centres in the UK and Ireland.

**Methods:**

A cross-sectional questionnaire, developed from literature, patient and public involvement and peer-reviewed for validity, was distributed via Bristol Online Survey to healthcare professionals in 29 tertiary paediatric centres. Recruitment used convenience sampling through social media and professional networks. Eligible participants gave electronic consent. Data was collected over 7 weeks (August–October 2022) and used descriptive analysis. Ethical approval was obtained from the University of Birmingham.

**Results:**

The response rate was 93% (27/29 centres). Of these, 41% reported having a PCCOT, predominantly nurse-led with notable growth since 2013. Team composition, size, funding models and training varied widely. Education and formal competencies were inconsistent, and many PCCOTs operated within incomplete governance systems often lacking process improvement functions. Commonly collected metrics included cardiorespiratory arrest rates, inpatient mortality and unplanned paediatric intensive care unit admissions.

**Conclusions:**

PCCOTs remain underdeveloped, with limited 24/7 coverage, inconsistent training and fragmented governance in comparison with adult CCOTs. Despite their critical role, most lack sustainable funding and robust evaluation frameworks. Newly developed paediatric-specific education standards now require implementation and impact assessment. National leadership, investment and standardisation are needed to ensure PCCOTs can deliver safe, effective and equitable care across the UK and Ireland.

**WHAT IS ALREADY KNOWN ON THIS TOPIC**

- Inadequate recognition and response to clinical deterioration contribute to preventable harm, with children, especially those with medical complexity, being at increased risk. While adult Critical Care Outreach Teams (CCOTs) are well-established, the role and impact of Paediatric CCOTs (PCCOTs) remain under-researched.

**WHAT THIS STUDY ADDS**

- This study provides the first national evaluation of PCCOTs across the UK and Ireland, revealing significant variability in service provision, training and governance. It highlights gaps such as inconsistent service coverage and lack of standardised frameworks, which affects service quality and sustainability.

**HOW THIS STUDY MIGHT AFFECT RESEARCH, PRACTICE OR POLICY**

- This study has the potential to shape future research on standardised guidelines, training and outcome metrics for PCCOTs. It may influence clinical practice by promoting consistency in service delivery and drive policy changes for national standards, funding and integration of PCCOTs across the UK and Ireland.

## Introduction

 Inadequate recognition and response to early signs of critical illness contribute to around one-third of avoidable deaths in hospitals in the UK and Ireland.^1^ Children are at increased risk due to a tendency for physiological compensation prior to rapid deterioration. Delayed escalation to senior staff is particularly hazardous and can result in avoidable Paediatric Intensive Care Unit (PICU) admissions, morbidity and mortality.^2^ This risk is further magnified in the growing population of Children with Medical Complexity (CMC), whose fragility due to multiple chronic conditions heightens susceptibility to critical illness and adverse outcomes.^3 4^

In light of the heightened risks faced by children, particularly those with medical complexity, there has been a global effort over the past 25 years to improve early recognition and response to deterioration within the in-hospital setting.^5 6^ This has driven the development of specialised teams, Critical Care Outreach Teams (CCOTs) in the UK and Rapid Response Teams (RRTs) and Medical Emergency Teams (METs) internationally, designed to detect early deterioration and intervene swiftly, a critical function for the vulnerable CMC population.^5^,^7^ While these terms are sometimes used interchangeably, in some institutions, they may represent distinct stages of escalation, with RRTs more likely to include a medical presence. These multidisciplinary teams are now regarded as essential to hospital safety, enhancing care quality and often described as the ‘safety engine of the hospital’.^8^ Their national implementation has been recommended by UK government reports^59^,^11^ and professional bodies.^12^,^14^

**Figure 1:**
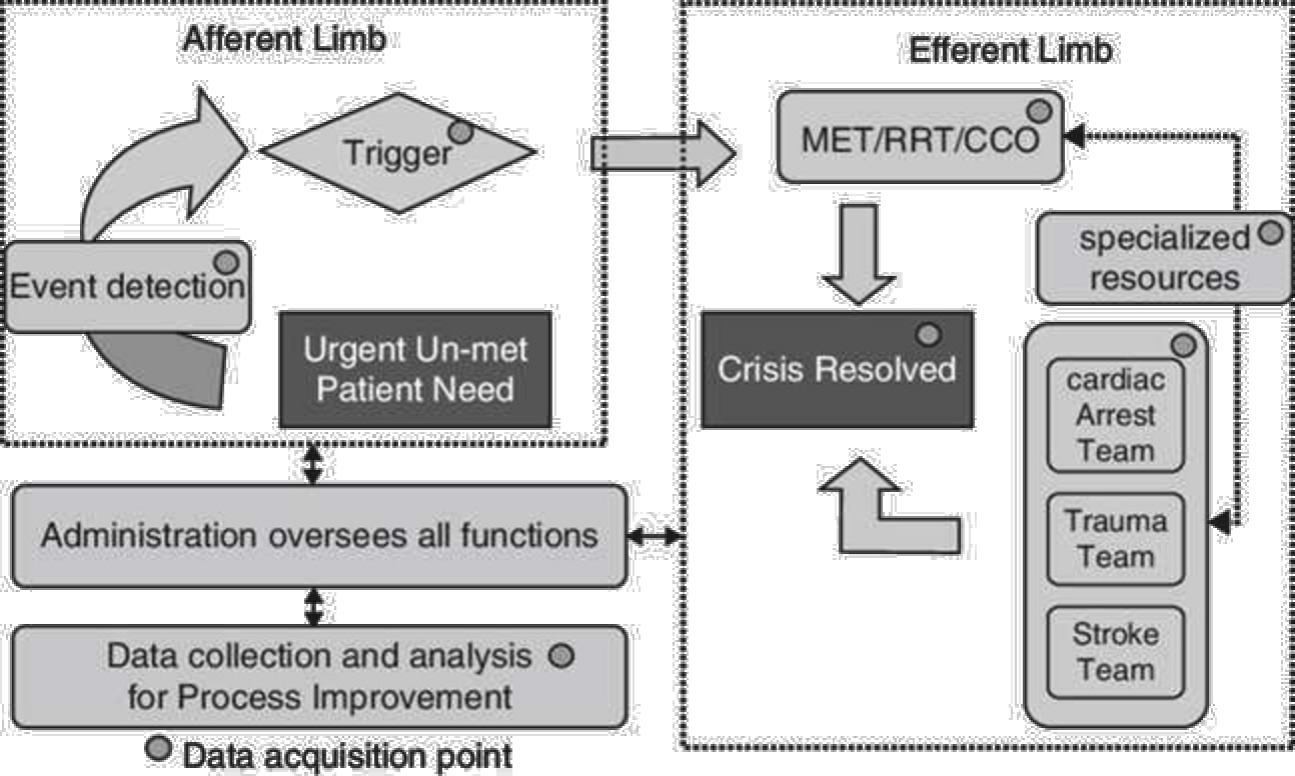
The rapid response system (RRS) (Rao *et al*, p. 26).^15^ CCO, critical care outreach; RRT, rapid response team.

The CCOT functions as a key component of the broader Rapid Response System (RRS), which comprises four interdependent elements: an afferent limb for early detection of clinical deterioration, CCOT as the efferent clinical response, a process improvement arm for evaluation and an administrative arm for oversight^15^ (figure 1). Together, these elements ensure timely intervention and help mitigate preventable morbidity and mortality.^15^

Within adult services, CCOTs are a well-established component of care, with 86% of acute National Health Service (NHS) hospitals in England reporting such services in 2021.^9^ Their structure, staffing and operations have been widely studied and note variation across hospitals.^12 15 16^ In contrast, the paediatric equivalent, Paediatric CCOTs (PCCOTs), remains under-examined. The recent Getting It Right First Time (GIRFT) report in Paediatric Critical are^10^ found that PCCOTs exist in only 43% (n=9) of tertiary paediatric centres in the UK and Ireland, yet no national study has systematically defined or mapped the current PCCOT landscape.

Given the depth of understanding available for adult CCOTs, the absence of equivalent data for PCCOTs highlights a clear disparity in the evidence base. Addressing this gap is essential to inform service development, enhance patient outcomes and support strategic planning.^17 18^ The aim of this study was to close this knowledge gap by determining the provision and characteristics of PCCOTs in tertiary paediatric centres across the UK and Ireland.

## Methods

### Questionnaire design and preparation

As no validated tool existed to collect the required data, a structured, cross-sectional questionnaire was developed based on a review of relevant literature, and in collaboration with a local Patient and Public Involvement and Engagement (PPIE) group. The questionnaire began with four preliminary items to confirm consent, determine hospital location and screen eligibility with the question: ‘Does your centre have a PCCOT?’. Centres that did not have a PCCOT were thanked for their participation, and the questionnaire was terminated. Respondents from centres with a PCCOT proceeded to complete the remaining 38 mandatory questions, which covered four key domains: demographics, PCCOT provision, team characteristics and quality metrics and governance.

The draft questionnaire underwent peer review by two registered healthcare professionals, one UK-based and one Europe-based, with expertise in questionnaire design, to ensure content validity and support response quality. It was then piloted two times with colleagues from the lead author’s PCCOT team. The first pilot (n=4) assessed question clarity, and the second (n=3) evaluated usability across different devices following transfer to the online platform Bristol Online Survey. Minor revisions to question format and content were made in response to both peer review and pilot feedback. The complete tool is available as online supplemental file 1.

### Patient and public involvement and engagement

The Young Persons Advisory Group (YPAG), a local PPIE group, was involved from the early stages of the study. Although the study focused on healthcare professionals, the group contributed by validating the relevance of the research topic from a public perspective and advised on the design and content of the questionnaire. They also provided input on recruitment strategies, particularly the use of social media, offering suggestions to improve clarity, engagement and accessibility. While they were not involved in the direct conduct of the study, the group was consulted on the anticipated burden and time commitment for participants and considered it acceptable. The YPAG was also engaged in dissemination planning, and study findings were fed back to the group in a format appropriate to their preferences.

### Participants and data collection

The target population comprised registered healthcare professionals from the 29 NHS paediatric tertiary centres across the UK and Ireland,^19^ with the objective of obtaining a single response per site from individuals knowledgeable about local PCCOT provision. Demographic details of the respondents are presented in the Results section. A convenience sampling strategy was used. Recruitment was conducted through a study poster shared via social media and distributed through the mailing lists of two professional networks: the Paediatric Critical Care Society Study Group (PCCS SG) and the National Outreach Forum (NOrF). Interested individuals contacted the research team directly via email to participate. The full study protocol, including the study poster and Participant Information Sheet (PIS), is provided in onlinesupplemental file 2ab.

Eligible participants, identified according to predefined inclusion criteria (table 1), were provided with a PIS and invited to a Microsoft Teams meeting to complete the consent process. During the meeting, eligibility was confirmed, study details were discussed and any queries were addressed. Informed electronic consent was then obtained, with the lead researcher witnessing the participant’s signature in accordance with Health Research Authority guidance for low-risk studies.^20^ Signed consent forms were returned via email. On receipt of consent, the questionnaire link was distributed via email and remained open for 7 weeks (August–October 2022). The study poster was re-circulated two times, at study weeks 5 and 7, to encourage participation. Following initial contact by potential participants, up to two follow-up reminder emails were sent, in accordance with the study’s ethical approval.

**Table 1:** Inclusion and exclusion criteria

Inclusion criteria	Exclusion criteria
Aged 18 years or older. Healthcare professionals currently working within a tertiary NHS hospital with a PICU in the UK and Ireland. Completion of the informed consent form (ICF).	Under 18 years of age. Healthcare professionals working in PICU not affiliated with the NHS. Professionals working in adult critical care outreach teams (CCOT) in the UK that review adult patients only. PCCOT services based outside the UK and Ireland. Incomplete ICF.

NHS, National Health Service; PCCOT, paediatric critical care outreach team; PICU, paediatric intensive care unit.

### Data analysis

The questionnaire was hosted on the Bristol Online Survey platform, and collected data were exported to Microsoft Excel 2016 for analysis. Descriptive statistics were used to summarise participant and service characteristics. No statistical software or inferential analysis was employed.^21^

### Data protection

Personally identifiable data, such as hospital affiliation, were collected but accessed only by the primary researcher, who acted as data custodian. Identifying details were replaced with pseudonyms, and data were stored securely using encrypted, password-protected systems. Only anonymised data were shared or uploaded to the University of Birmingham Research Data Store.

## Results

### Demographics of respondents

The questionnaire achieved a 93% participation rate, with participants representing 27 of the 29 paediatric tertiary NHS hospitals across all four nations of the UK and Ireland. Respondents were recruited via PCCS SG (n=12), social media (n=7), inter-colleague sharing (n=6) and NOrF (n=2). Occupations included medical consultants (n=11), PCCOT members (n=8), PICU nurses (n=2), clinical nurse educators (n=2) and single responses from a chief nurse, an emergency department nurse, a PICU registrar and a resuscitation nurse.

### PCCOT provision

**Figure 2:**
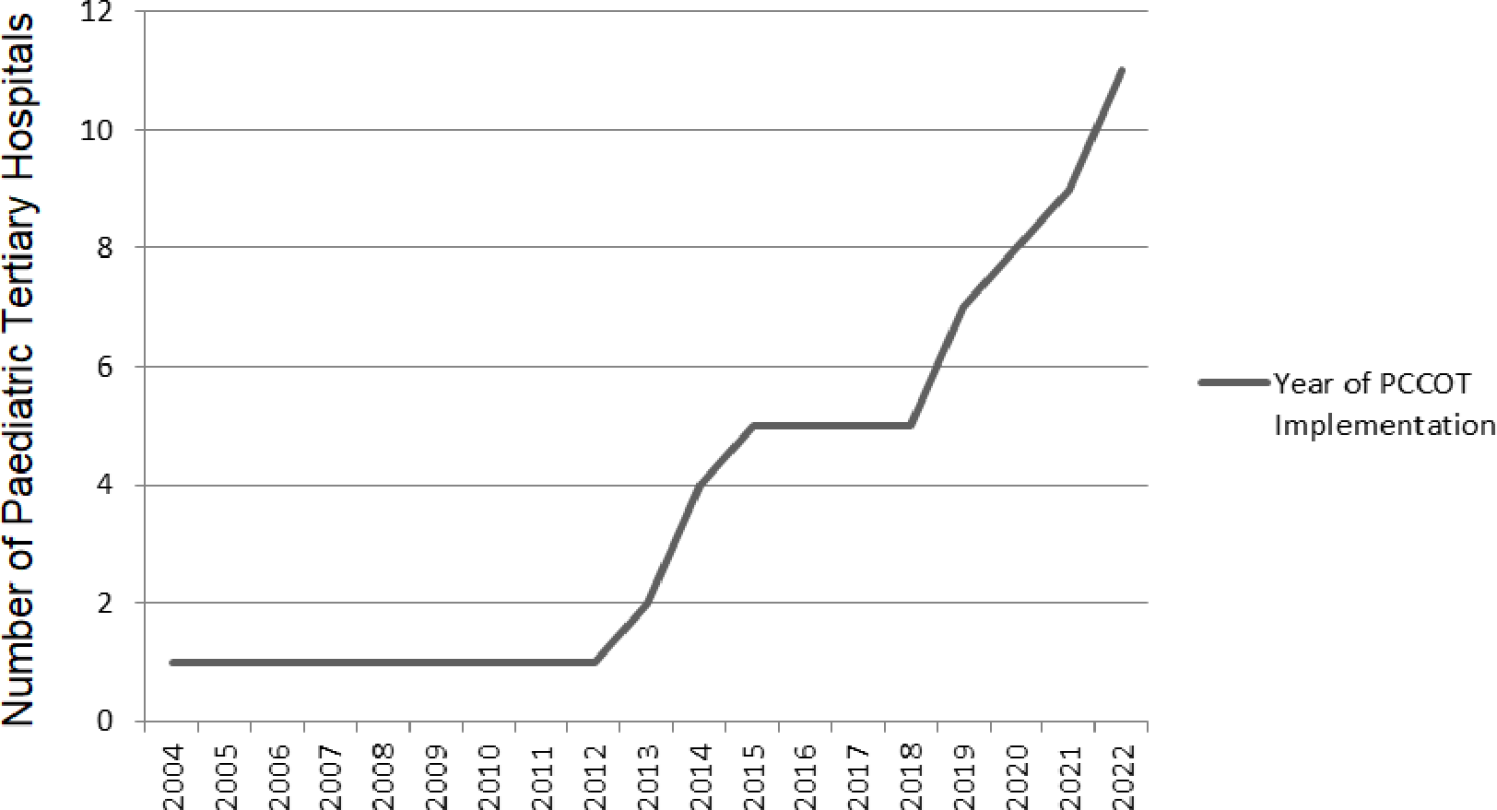
Cumulative number of paediatric tertiary hospitals with a PCCOT service by year. PCCOT, paediatric critical care outreach team.

**Figure 3:**
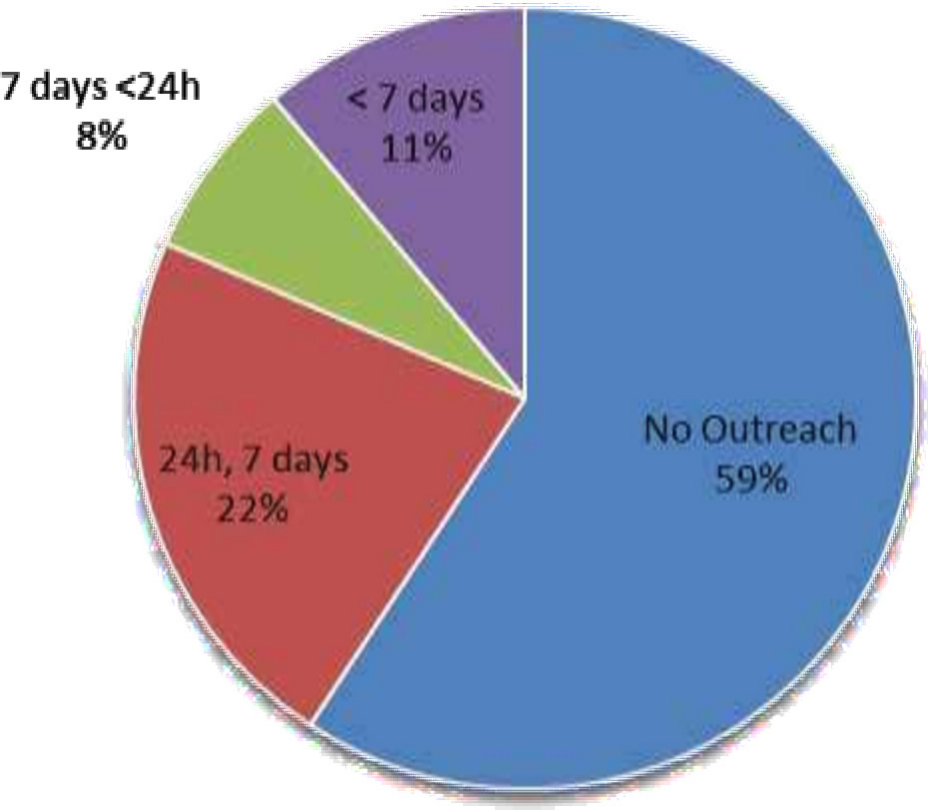
Availability of PCCOT services in paediatric tertiary centres in the UK and Ireland. PCCOT, paediatric critical care outreach team.

Of the 27 responding paediatric tertiary centres, 41% (n=11) reported a PCCOT, located in centres in England (n=10) and Scotland (n=1). A further 11% (n=3) indicated that they were in the process of developing a business case to establish a service. All established PCCOT services were in major trauma centres with neurosurgery, neurology, high dependency care and long-term ventilation provision (table 2). The first PCCOT started in 2004, with exponential growth from 2013 (figure 2). Of the 11 PCCOT services, over 55% (n=6) operated 24/7, while the rest had variable availability (figure 3). Funding sources included PICU (n=4), hospital at night service (n=3) and single sources from PICU interhospital transport service (n=1), idiopathic spinal pathway (n=1), winter surge (n=1) and missing data (n=1).

**Table 2:** Demographics of sites with PCCOTs

	Sites with a PCCOT
Sample size	11
Region, n (%)	
England	10 (91)
Ireland	0
Northern Ireland	0
Wales	0
Scotland	1 (9)
Hospital types	
Specialist paediatric hospital	4 (36)
Mixed specialist hospital for adult and paediatrics	7 (64)
Co-located services	
Paediatric cardiac intensive care unit	6 (54)
Extracorporeal membrane oxygenation	5 (45)
Haematology	10 (91)
High dependency unit	11 (100)
Long-term ventilation	11 (100)
Major trauma centre	11 (100)
Neurology	11 (100)
Neurosurgery	11 (100)
Palliative care	9 (82)
Oncology	10 (91)
Retrieval service	8 (73)

PCCOT, paediatric critical care outreach team.

### PCCOT characteristics

All teams were mono-professional, consisting exclusively of either nurses or medical staff. The majority of PCCOTs (82%, n=9) were nurse-led, while a smaller proportion (18%, n=2) were led and staffed by medical consultants. The median team size was 8 members (range: 3–11), with most services (82%, n=9) operating with a single practitioner on duty per shift. Nurse-led teams varied in composition, including nurses at different seniority levels based on the UK NHS pay and responsibility banding system: mid-level registered nurses (equivalent to Band 6; 33%, n=3), senior clinical nurses (Band 7; 22%, n=2), a combination of Band 6 and Band 7 nurses (33%, n=3) and Advanced Clinical Practitioners (ACPs) (Band 8a; 11%, n=1). Leadership roles were most commonly held by Band 7 nurses (67%, n=6), with the remainder led by Band 8a nurses (33%, n=3).

**Figure 4:**
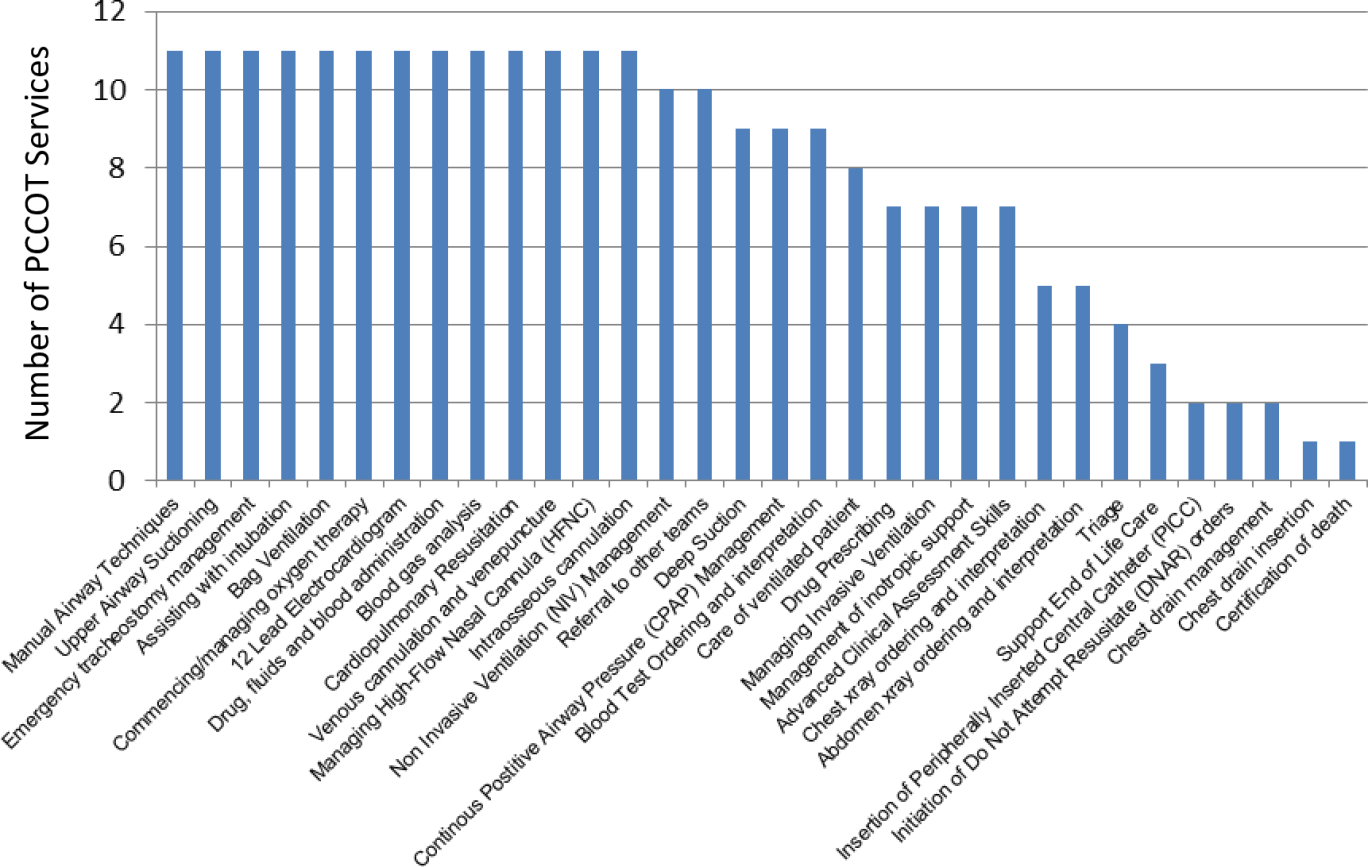
Reported PCCOT skillset. PCCOT, paediatric critical care outreach team.

Referrals to PCCOTs were primarily for respiratory concerns, including distress, abnormal respiratory rate and hypoxia, and were received via face-to-face request (n=9), bleep (n=9) or phone (n=8). PCCOT practitioners reported a broad clinical skillset encompassing paediatric airway and respiratory management, vascular access, drug administration and prescribing (figure 4). More than half of participants reported undertaking proactive rounds (n=4) or having a watch list of children at risk of deterioration (n=5).

The provision of education and training across PCCOT services demonstrated considerable variability. Overall, 67% (n=7) of teams reported delivering education to ward-based staff; in the majority of these cases (n=6), this was provided informally through real-time feedback and debriefing during PCCOT activations. Only one service reported offering a formal education programme focused on the recognition and management of clinically deteriorating patients. Furthermore, opportunities for education and professional development among PCCOT team members were relatively limited. Regular team meetings were reported as a means of training in 45% (n=5) of services, while 36% (n=4) engaged in scenario-based training. The implementation of structured competency frameworks specific to PCCOT practice was reported in only 18% (n=2) of teams.

### PCCOT quality metrics and governance

All PCCOT teams reported having functional afferent and efferent limbs of the RRS, which are essential for early detection and clinical response (figure 1). However, the other two key components necessary for a comprehensive RRS, the process improvement and administrative arms, were often missing. Specifically, 45% of teams (n=5) lacked a process improvement component, and 73% (n=8) did not have an administrative arm. Consequently, no service had a fully established RRS encompassing all four elements. Only three outcome metrics were consistently collected across all PCCOT centres: cardiorespiratory arrest rate, inpatient mortality and unplanned PICU admissions.

## Discussion

### PCCOT provision

In the Autumn of 2022, 41% (n=11) of tertiary paediatric hospitals in the UK and Ireland had an established PCCOT service. This compares with 86% of adult acute NHS hospitals reporting CCOT provision, as noted in the GIRFT report on adult critical care^9^. In England, PCCOTs were first established in 2004, with limited early adoption followed by broader implementation from 2013, whereas adult CCOTs expanded rapidly between 2000 and 2002.^22^ The broader and earlier integration of adult CCOTs likely reflects the longer-standing presence of adult Intensive Care Units (ICU) in the UK, which began developing in the late 1960s.^23^ This has supported the growth of a more mature infrastructure, stronger research base and clearer clinical pathways for deterioration management.^23 24^

In contrast, PCCOT provision remains limited, likely due to the later development of PICU services in the early 1990s, smaller and more diverse patient populations and the added complexity of managing children, especially the CMC population.^3^,^525^ Most PCCOTs were based in English centres (91%, n=10), consistent with national activity as England accounted for 77.3% of PICU admissions across the UK between 2021 and 2023.^26^ This geographic concentration not only reflects the demand for PCCOTs in high-volume centres but also underscores potential disparities in service availability across the UK and Ireland. Since this study was conducted in 2022, management of deteriorating paediatric patients in England has evolved with the introduction of the inpatient National Paediatric Early Warning Score and Martha’s Rule,^6^ both requiring PCCOT support, reinforcing the relevance of the study’s findings.

Despite the growing demand, full compliance with national guidance remains limited. National guidance recommends that PCCOTs operate 24 hours a day, 7 days a week (24/7)^9 10^; however, only 22% (n=6) of paediatric tertiary centres currently meet this standard. Compliance is similarly poor in adult CCOTs, with only 42% reporting 24/7 coverage.^9^ A likely contributing factor is the lack of dedicated and sustainable funding, as highlighted in the literature.^27^ Our study found PCCOTs are financed through varied and sometimes temporary sources, such as winter surge funding, raising concerns about long-term viability. There is a scarcity of literature addressing responsibility for funding outreach services, and inconsistent funding has been identified as a barrier to service delivery and development.^27 28^ While this study did not identify any discontinued PCCOT services, reports have linked the cessation of adult CCOTs to inadequate financial resources.^22^

### PCCOT characteristics

The identified financial, geographical and operational challenges likely contribute to the observed variation in the size and composition of PCCOT workforces. This study found that PCCOT services are predominantly nurse-led and structured as mono-professional teams, a model also seen in UK adult CCOT.^22^ In contrast, international RRTs are typically multidisciplinary.^29^ Both UK PCCOTs and adult CCOTs are primarily staffed with Bands 6 and 7 nurses, highlighting a continued reliance on nursing staff for UK CCOT and PCCOT provision.^11 22 30^ ACP involvement in PCCOTs was notably low at 11% (n=1), compared with 35% in adult CCOTs,^22^ despite international evidence emphasising the vital role of ACPs in clinical decision-making, ICU transitions and interdisciplinary collaboration in CCOT services.^31^ While local resource-driven models have shaped staffing and service delivery,^5 22 28^ national minimum specifications may be required to standardise CCOT and PCCOT provision and characteristics.^32^

Reflecting this variability, this study found that PCCOT skills are diverse, with a primary focus on the assessment, diagnosis and treatment of deteriorating children, particularly relevant given the paediatric population’s heightened risk of respiratory compromise.^3 4 33^ Most PCCOTs reported undertaking proactive strategies, such as rounding and the use of watcher lists, to support early identification of at-risk patients, aligning with research that highlights the increased need for continuous surveillance in paediatric settings compared with adult CCOT services.^34^ Despite the specialised demands of PCCOT roles, this study identified limited access to team meetings, scenario-based training and formal competencies, highlighting the need for structured education to enhance clinical capability.^31 35^ National competencies published in 2022^8^ at enhanced and advanced levels, following the completion of this study, provide a foundation for standardised practice and role development, warranting evaluation as they become embedded in practice.

### PCCOT quality metrics and governance

However, education alone is insufficient without structural support. All PCCOT services in this study were found to operate within an incomplete RRS, a finding consistent with existing literature.^29 35^ An incomplete RRS poses significant risks, particularly the disconnection between its component parts, which can hinder effective service evaluation, quality assurance and clinical governance.^24 29 36^ Notably, two of the three most commonly collected metrics, mortality rate and ICU admission rate, have been excluded from recent national guidance^12^,^14^ despite being previously recommended.^5 11^ This shift reflects longstanding challenges in demonstrating consistent reductions in these outcomes following the implementation of CCOTs, as reported across multiple studies.^37^,^39^

As a result, both CCOT and PCCOT services often lack robust evaluation frameworks, a limitation widely acknowledged in the literature and attributed to the inherent complexity of CCOT and PCCOT, highlighting the need for more appropriate and objective measures of impact.^18 32 40^ Future research should prioritise the development of a definitive core metric dataset tailored to PCCOT, with consideration given to the value of formal endorsement or potential inclusion within the National Clinical Audit and Patient Outcomes Programme. As an interim approach, linking PCCOTs to a hospital-wide Deteriorating Patient Working Group, as recommended by the paediatric critical care GIRFT report,^10^ may strengthen clinical governance oversight. Future editions of relevant professional standards, such as those from the PCCS and NOrF, should provide clearer guidance on PCCOT configuration and governance arrangements.

### Strengths and weaknesses

This study is the first national exploration of PCCOT characteristics in the UK and Ireland, offering novel insights into a service area with limited prior research. A major strength is the high response rate (93%), with 27 of 29 tertiary paediatric centres participating. The findings also align with existing research in adult CCOTs, particularly around variability in service models, team composition and governance, reinforcing broader trends across Critical Care Outreach. However, a key limitation is the self-reported nature of the data, which may be influenced by recall bias or inconsistent interpretation across sites. Despite this, the consistency of responses and the national scope provide a robust foundation for further research and service development.

## Conclusion

This study reveals significant variability and persistent challenges in the provision and development of PCCOTs across tertiary centres in the UK and Ireland. Despite their essential role in managing deteriorating and complex children, only 41% of responding paediatric tertiary hospitals reported having a PCCOT, most of which are nurse-led and operate without stable funding or robust governance. These services lag behind adult CCOTs in both scale and support and continue to rely on outdated outcome metrics that no longer align with national recommendations.

Although recent progress has been made with the development of paediatric-specific standardised education frameworks, their implementation and impact remain unassessed. Most PCCOTs operate within incomplete RRS, further highlighting the disconnect between national policy and local practice. These findings underscore the urgent need for national oversight, including a minimum service specification, secure funding models and validated outcome measures tailored to paediatric care. PCCOTs make a critical contribution to paediatric patient safety and timely escalation of care. Measures are required at both local and national levels: linking PCCOTs to hospital-wide Deteriorating Patient Groups may strengthen local governance, while professional standards should provide clearer guidance on PCCOT configuration and oversight. However, without strategic investment and formal evaluation of emerging educational and governance structures, the sustainability, effectiveness and future development of PCCOTs will remain at risk.

## Supplementary material

10.1136/bmjpo-2025-003920 online supplemental file 1

10.1136/bmjpo-2025-003920 online supplemental file 2

10.1136/bmjpo-2025-003920 online supplemental file 3

## Data Availability

Data may be obtained from a third party and are not publicly available. All data relevant to the study are included in the article or uploaded as supplementary information.
