# Supplementary material for: Exploring the provision and structure of paediatric critical care outreach teams (PCCOTs) in the UK and Ireland: a national questionnaire study
Source: BMJ Paediatr Open. 2025 Dec 21;9(1):e003920. doi: 10.1136/bmjpo-2025-003920 (PMC12718590; doi:10.1136/bmjpo-2025-003920)
Supplement: online supplemental file 1 [file bmjpo-9-1-s001.docx]

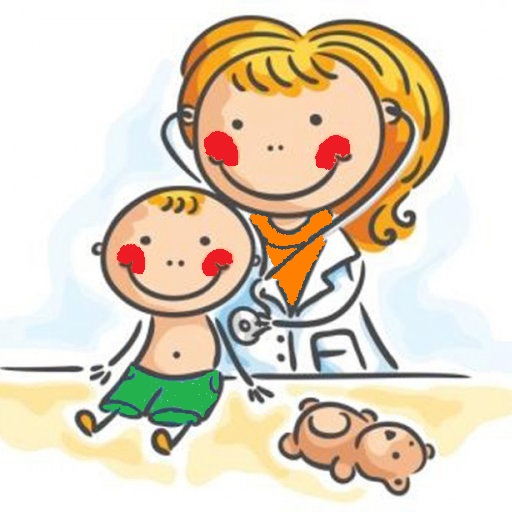


Study: **D**iscovery of **I**deal **V**ariables and **E**xcellence markers in paediatric **R**apid **R**esponse **T**eams (DIVERRT)

Participant study ID:

**Data Collection Tool: Questionnaire**

| **First question** | | **Potential answers** |  |  |
| --- | --- | --- | --- | --- |
| 1 | Have you read the participant information sheet and are you happy to continue? | Yes/No |  |  |
| 2 | Role of person completing the questionnaire | Lead of RRT  Member of RRT  Lead Resuscitation Officer  Other (freetext)  Rather not say |  |  |
| 3 | Please indicate the location of your hospital | 1. Addenbrooke's Hospital, Cambridge 2. Alder Hey Children’s Hospital, Liverpool 3. Birmingham Children’s Hospital 4. Bristol Royal Hospital for Children 5. Children’s Health Ireland at Crumlin, Dublin 6. Children’s Health Ireland at Temple Street, Dublin 7. Evelina London Children’s Hospital 8. Freeman Hospital, Newcastle upon Tyne 9. Glenfield Hospital, Leicester 10. Great Ormond Street Hospital, London (PICU/NICU/CICU) 11. Great North Children’s Hospital, Newcastle upon Tyne 12. James Cook University Hospital, Middlesbrough 13. John Radcliffe Hospital, Oxford 14. King’s College Hospital, London 15. Leeds General Infirmary 16. Leicester Royal Infirmary 17. Noah’s Ark Children’s Hospital for Wales, Cardiff 18. Nottingham Children’s Hospital 19. Royal Belfast Hospital for Sick Children 20. Royal Brompton Hospital, London 21. Royal Hospital for Children, Glasgow 22. Royal Hospital for Sick Children, Edinburgh 23. Royal Manchester Children’s Hospital 24. Royal Stoke University Hospital 25. Sheffield Children’s Hospital 26. Southampton Children’s Hospital 27. St Mary’s Hospital, London 28. St George’s Hospital, London 29. The Royal London Hospital 30. Other (free text)   Rather not say |  |  |
| **Screening questions** | | |  |  |
| 4 | Does your hospital have a Cardiac Arrest Team or equivalent? | Yes/No/Rather not say |  |  |
| 5 | A Rapid Response Team (RRT) are a group of professionals with an advanced skillset that are activated on the identification of a deteriorating patient. An alternative name for a RRT is a Critical Care Outreach Team, a Patient at Risk Team or Medical Emergency Team.  Does your hospital have a RRT or equivalent? | Yes/No |  |  |
| **Demographics** | | |  |  |
| 6 | Does your RRT care for adult patients, paediatric patients or both? | Adult Patients only  Adult Patients mostly but responsible for responding to paediatric collapse/ cardiac arrest alert  Paediatrics Patient only  Paediatric Patients mostly but responsible for responding to adult paediatric collapse/ cardiac arrest alert  Both adult and paediatric patients  Rather not say |  |  |
| 7 | Which descriptor best describes your hospital? | District General Hospital  Specialist Hospital - Adult  Specialist Hospital - Paediatric  Other (free text)  Rather not say |  |  |
| 8 | Please indicate the number of admissions to your hospital per year | Scroll bar |  |  |
| 9 | Please indicate the number of inpatient beds in your hospital | Scroll bar |  |  |
| 10 | Please indicate the average number of beds available on each ward in your hospital.  Please note, if your hospital does not the described ward, please leave the scroll bar at zero. | Paediatric Intensive Care Unit/Level 3 Paediatric Critical Care Unit (scrollbar)  Paediatric Cardiac Intensive Care Unit (scrollbar)  Paediatric High Dependency Unit/Level 2 Paediatric Critical Care Unit  Neonatal Intensive Care Unit (scrollbar)  Neonatal High Dependency Unit (scrollbar)  Adult Intensive Care Unit (scrollbar)  Adult High Dependency Unit (scrollbar)  No Intensive Care or high dependency unit (scrollbar)  Other (freetext)  Rather not say |  |  |
| 11 | What specialities and nationally commissioned services for paediatric are there within your hospital? Please check all that apply. | Extra Corporeal Membrane Oxygenation (ECMO)  Paediatric Intensive Care Unit retrieval/transport Service  Neonatal Intensive Care Unit retrieval/transport Service  Paediatric Long Term Ventilation  Paediatric Congenital Heart Disease Services   - Level 1 Specialist Surgical Centre - Level 2 Specialist Cardiology Centre - Level 3 Local Cardiology Centre   Paediatric Medicine   - Endocrinology and Diabetes - Gastroenterology, Hepatology and Nutrition - General Paediatrics - Haematology - Metabolic Disorders - Neurology - Oncology - Level 1 Paediatric Oncology Shared Care Service - Level 2 Paediatric Oncology Shared Care Service - Level 3 Paediatric Oncology Shared Care Service - Palliative Care - Renal - Respiratory - Rheumatology   Paediatric Surgery   - Cleft and lip services - Craniofacial services - Ear, Nose and Throat (ENT) - Epilepsy surgery services - General surgery - Neurosurgery - Urology   Specialist Services   - Major Trauma Centre - Specialist centre for burn injuries - Specialist liver disease service   Transplantation Centre   - Liver - Small bowel - Cardiothoracic - Other (free text) |  |  |
| **Response to deteriorating patient** | | |  |  |
| 12 | Do you have a policy within your hospital which clearly defines the process for escalation of a deteriorating patient? | Y/N/rather not say |  |  |
| 13 | Is compliance with this policy audited in your hospital? | Yes – by my team  Yes – not by my team  Unsure  No  Rather not say |  |  |
| 14 | By which method are deteriorating patient detected in your hospital? Please all that apply. | 1. Identification of patients at risk of deterioration on hospital admission using a tool, 2. Routine ward rounds by RRT team to identify patients at risk, 3. Review of all inpatient admissions by RRT to identify patient at risk of deterioration 4. Paediatric Early Warning Score (PEWS)/track and trigger system 5. Single observation trigger system 6. Professional concern 7. Parental concern/referral 8. Automated/automatic referral to RRT 9. Rather not say |  |  |
| 15 | How are **ward nurses** prepared to identify, respond to and escalate concerns regarding the deteriorating patient in your hospital? Please check all that apply. | Clinical experience  Competencies/formal assessment on ability to recognise a deteriorating patient and escalate  Feedback and debrief from deterioration events with individual staff members  Feedback of learning from RRT events and actions to all  Frequently repeating education program  Shadowing of RRT by ward nurses  Simulation training  One time educational program – trust wide (such as HDU course)  One time educational program – on individual wards  Real time education by RRT following activation call  Specific training on the escalation policy  Ward link workers with RRT  Other – free text  Unsure  Nurses receive no additional training  Rather not say |  |  |
| 16 | How are **ward doctors** prepared to identify, respond to and escalate concerns regarding the deteriorating patient in your hospital? Please check all that apply. | Clinical experience  Competencies/formal assessment on ability to recognise a deteriorating patient and escalate  Feedback and debrief from deterioration events with individual staff members  Feedback of learning from RRT events and actions to all  Frequently repeating education program  Shadowing of RRT by ward nurses  Simulation training  One time educational program – trust wide (such as trust induction)  One time educational program – on individual wards  Real time education by RRT following activation call  Specific training on the escalation policy  Other – free text  Unsure  Doctors receive no additional training  Rather not say |  |  |
| 17 | Are your RRT involved in the training given to the ward based team? | Yes – for both ward doctors and nurses  Yes – for doctors only  Yes – for nurses only  No – for neither ward doctors nor nurses  Unsure  Rather not say |  |  |
| **RRT Service** | |  |  |  |
| 18 | What is the name of the RRT in your hospital ? | Freetext  Rather not say |  |  |
| 19 | What year was the RRT service set up? | Scrollbar |  |  |
| 20 | Why was the RRT set up in your hospital? | Clinical need,  In response to clinical incidence,  Other (free text)  Rather not say |  |  |
| 21 | Which hospital service is your RRT connected to? | Paediatric Intensive Care Unit  High Dependency Care Unit  Emergency Department  Theatres  General Paediatrics  Hospital at Night Service  Other (freetext)  Rather not say |  |  |
| 22 | What hours does your RRT service operate? | 24 hours a day and 7 days a week  Other (free text)  Rather not say |  |  |
| 23 | How are referrals made to the RRT in your hospital? Please check all that apply. | Via switch  Via bleep  Via computer  Via phone  Face to face  Other (free text)  Rather not say |  |  |
| 24 | How many referrals has your RRT received in the last year? | Scroll bar  Data not collected  Rather not say |  |  |
| 25 | On a scale of 1 to 12, where 12 is not seen or seen very irregularly and 1 is seen commonly, how would you rate the referral reasons to your service?  There will be a roller bar by each question | Airway compromise  Tachypnoea/Bradypnoea  Respiratory Distress  Dyspnoea  Hypoxia  Tachycardia  Bradycardia/Hypotension  Arrhythmia  Sepsis  Altered conscious state  Hypoglycaemia  Reduced urinary output |  |  |
| 26 | Are there any referral reasons you see in your service not included in the question above? If yes, please advise in the text box. | Freetext  Rather not say |  |  |
| RRT Team Composition | |  |  |  |
| 27 | Please state the numbers of professionals within your hospital’s RRT | Nurse band 5 (scroll bar for numbers)  Nurse band 6 (scroll bar)  Nurse band 7 (scroll bar)  Nurse band 8a (scroll bar)  Doctor – foundation (scroll bar)  Doctor - speciality registrar (scroll bar)  Doctor - consultant/other (free text)  Physician Associate (PA) band 6 (scroll bar)  Physician Associate (PA) band 7 (scroll bar)  Physician Associate (PA) band 8a (scroll bar)  Physiotherapist band 5 (scroll bar)  Physiotherapist band 6 (scroll bar)  Physiotherapist band 7 (scroll bar)  Physiotherapist band 8a (scroll bar)  Pharmacist (scroll bar)  Other profession – free text  Rather not say |  |  |
| 28 | How many staff work per shift for your hospital’s RRT? | 1 professional per shift  2 professionals per shift  More than 2 professionals per shift  Rather not say |  |  |
| 29 | Which of the following skills are your hospital’s RRT members competent in? Please check all that apply. | Rather not say  Airway   - Manual airway techniques - Suctioning upper airway - Deep suction - Insertion of airway (guedel and nasopharyngeal) - Insertion of laryngeal mask airway (LMA) - Management of blocked or displaced tracheostomy - Assisting with endotracheal intubation - Performing endotracheal intubation   Breathing   - Anaesthetic bag ventilation - Bag Valve Mask ventilation - Commencing and managing oxygen therapy - Commencing and managing High Flow Nasal Cannula (HFNC) therapy - Commencing Continuous Positive Airway Pressure (CPAP) therapy via mask - Commencing and managing Non Invasive Ventilation therapy via mask - Commencing and managing Invasive Ventilation (after intubation) - Care of a ventilated patient - Chest drain insertion   Circulation   - 12 lead electrocardiogram - Arterial blood gas - Blood/blood product administration - Blood/blood product - requesting - Blood/blood product – prescribing - Blood gas analysis - Cardiopulmonary Resuscitation - Capillary blood gas - Cardioversion - Defibrillation - Drug administration - Drug prescribing - Fluid bolus - administration - Fluid bolus - prescribing - Insertion of arterial line - Insertion of central venous line - Insertion of peripherally inserted central catheter (PICC) - Management of invasive haemodynamic monitoring - Management of inotropic support - Venepuncture - Venous cannulation   Assessment, Investigations and decisions   - Abdomen x ray interpretation - Abdomen x ray ordering - Advanced clinical assessment and diagnosis - Blood test interpretation - Blood test ordering - Certification of death - Chest x ray interpretation - Chest x ray ordering - Commencing initial therapy - Initiating Do Not Attempt Resuscitation order or End of Life Care - Interhospital transfer - Intrahospital transfer - Intraosseous needle insertion - Referral to another healthcare professional - Triage - Other (free text) |  |  |
| 30 | What training and education occurs within your RRT? Please check all that apply. | Advanced Clinical Practice (MSc)  Advanced Communication training  Advanced Decision making course  Advanced Diagnostic Skills  Advanced Examination course  Advanced Life Support (ALS) (Adult)  ALS (Adult) – faculty member  Advanced Paediatric Life Support (APLS)  APLS – faculty member  Attendance at national conference  Attendance at international conference  Basic life support (BLS)  BLS – trainer  Critical Care programmes feeding back RRT events and actions  Formal education qualification  Program reflecting and feeding back RRT events and actions  Fundamental Critical Care Support Course  High fidelity simulation training  Leadership and management course  ‘Leading a debrief’ training  Mock arrest calls  Mock trauma calls  Non-Technical Skills training  Prescribing – non-medical  Prescribing – medical  Paediatric immediate life support (PILS)  Paediatric Critical Care Level 2 (previously High Dependency Unit) course  Paediatric Critical Care Level - foundations course  Paediatric Critical Care Level 3 – specialist course  Research and audit qualification  Team meeting  Team training – from outside professionals  Team training – from other members of RRT  Situational Awareness training  Specialised training to manage Critical Incidents  Team working and co-operation training  Other  Rather not say |  |  |
| 31 | How do RRT members have their clinical and non- clinical performance evaluated? Please check all that apply. | Ad hoc  Appraisal with line manager  Checklist completion during clinical practice/simulation  Debrief following simulation training or significant clinical events  Feedback from other professionals  Feedback from patients and parents  Following complaints/expression of concern  Self-rating  Use of a rating scale during clinical practice/simulation  Unsure  Other (freetext)  Rather not say |  |  |
| **Scenario 1** | | |  |  |
| 32 | Scenario 1: Your hospital’s RRT receives a call for **patient deterioration.** The ward nurse is concerned that the six month old infant in her care has an increased respiratory rate, moderate work of breathing and is needing increasing amounts of inspired oxygen to maintain adequate oxygen saturations**.**  How many professionals respond to the call? | Roller bar  Free text  Rather not say |  |  |
| 33 | Do any other professionals, other than the RRT, respond in this scenario? If so, what are the roles of the responders? | Y/N  Freetext (only activates if says yes)  Rather not say |  |  |
| 34 | Who is the leader in this scenario? | RRT nurse  RRT doctor  RRT Physician Associate  RRT Physiotherapist  PICU doctor  PICU advanced clinical practitioner  Lead medic/Regional Medical Officer (RMO)  Other (freetext)  Rather not say |  |  |
| 35 | After examination, you decide the patient requires escalation of care to PICU? Who do you contact to facilitate this? | - PICU nurse in charge for a bed – RRT team have PICU admission rights - PICU trainee doctor or advanced clinical practitioner for patient review and decision to admit to PICU - PICU consultant for patient review and decision to admit to PICU - Other (freetext) - Rather not say |  |  |
| 36 | Do RRT staff members have responsibilities elsewhere in the hospital? If so, what are the other responsibilities? | Y/N  Freetext (only activates if says yes)  Rather not say |  |  |
| **Scenario 2** | | |  |  |
| 37 | Scenario 2: Your hospital’s RRT receives a referral for inpatient paediatric **cardiorespiratory arrest**, how many professionals of each role respond to the call? Please select the number of each professional. | Unsure – RRT is not a member of the cardiorespiratory arrest team  Rather not say  RRT (number scroll bar)  Ward nurse (number scroll bar)  PICU consultant (number scroll bar)  PICU speciality registrar (number scroll bar)  PICU advanced clinical practitioner (number scroll bar)  PICU nursing team (number scroll bar)  General Medicine consultant (number scroll bar)  General Medicine registrar/Regional Medical Officer (RMO) (number scroll bar)  General Medicine junior doctor (number scroll bar)  Anaesthetic consultant (number scroll bar)  Anaesthetic registrar (number scroll bar)  Theatre Staff Member (number scroll bar)  Pharmacist (number scroll bar)  Porter (number scroll bar)  Hospital Resuscitation Officer  Patient parent team – junior doctor (number scroll bar)  Patient parent team – registrar (number scroll bar)  Patient parent team – consultant (number scroll bar)  Unsure  Other (freetext) |  |  |
| 38 | Who is the team leader in this scenario? | Rather not say  Unsure – RRT is not a member of the cardiorespiratory arrest team  Member of RRT  Ward nurse  PICU consultant  PICU speciality registrar  PICU advanced clinical practitioner  PICU nursing team  General Medicine consultant  General Medicine registrar/Regional Medical Officer (RMO)  General Medicine junior doctor  Anaesthetic consultant  Anaesthetic registrar  Theatre Staff Member  Pharmacist  Porter  Hospital Resuscitation Officer  Patient parent team – junior doctor  Patient parent team – registrar  Patient parent team – consultant  Unsure  Other (freetext) |  |  |
| **Other roles** | | |  |  |
| 39 | What other roles does your RRT perform within the hospital? | Rather not say  Avoiding unplanned admissions to PICU  Avoiding unplanned readmissions to PICU following discharge  Clinical Governance  Commencing discussion regarding treatment limitation and ‘Do not attempt cardiopulmonary resuscitation’ orders  Educational support to ward staff  Liaison between teams  Mentor  Member of trauma team  Patient review following PICU discharge  Patient safety  PICU follow up post hospital discharge  Practical support to ward staff  Provide clinical expertise  Psychological support to ward staff  Supporting PICU discharge  Teacher  Other (free text) |  |  |
| **Quality Metrics** | | |  |  |
| 40 | Which of the following quality metrics are collected in your hospital? Please check all that apply. | 1. Adverse Event Rate 2. Inpatient Mortality 3. Hospital Standardised Mortality Ratio (HSMR) 4. 28/30 day mortality 5. Cardiorespiratory arrest rate 6. Hospital Length of Stay 7. Parent/family activation 8. Automated RRT activation 9. Delayed RRT activation rate 10. Missed RRT activation rate 11. Number of inappropriate RRT activations 12. Outcomes of RRT activation: 13. Stay on ward 14. Commence Do Not Resuscitate Order or End of Life Care 15. Unplanned transfer to High Dependency Care 16. Unplanned transfer to Intensive Care Unit 17. Inpatient Mortality 18. RRT response time 19. Critical Care interventions during RRT call 20. RRT documentation of written treatment goals 21. Mean duration of RRT calls 22. RRT user satisfaction 23. Review of learning from each RRT activation 24. Time to ICU admission from first concern/abnormal parameter 25. Severity of illness or critical deterioration in ICU 26. ICU outcome 27. ICU Length of stay 28. Time of discharge from ICU 29. Post ICU discharge by RRT 30. Time between ICU discharge and RRT review 31. Period of post ICU follow up 32. Unplanned ICU readmission 33. Medium term patient outcomes 34. Follow up after patient discharge 35. Unplanned hospital readmission 36. Long term patient outcomes 37. Cost effectiveness of RRT 38. Rather not say |  |  |
|  | **Final questions** |  |  |  |
| 41 | Would you be happy to be contacted to participate in semi structured interview via zoom regarding the answers in this questionnaire? This would be undertaken at a convenient time for you. | Yes/no |  |  |
| 42 | If so, please provide your consent for the storage of your contact details by clicking **this link** (please open in a new window) | **Add Hyperlink** |  |  |
| 43 | Would you like to be advised to the findings of this study once it is completed?  This would comprise of an emailed invite to attend a presentation on the study outcomes | Yes/no |  |  |
|  | If so, please provide your email address. | Free text |  |  |
|  | Thank you for your participation. |  |  |  |
