# Supplementary material for: Exploring the provision and structure of paediatric critical care outreach teams (PCCOTs) in the UK and Ireland: a national questionnaire study
Source: BMJ Paediatr Open. 2025 Dec 21;9(1):e003920. doi: 10.1136/bmjpo-2025-003920 (PMC12718590; doi:10.1136/bmjpo-2025-003920)
Supplement: online supplemental file 2 [file bmjpo-9-1-s002.docx]

## Full/long title of the study

Evaluation of the optimal components and quality metrics of Rapid Response Teams for the management of critically ill children in a specialist children’s hospital.

## Short study title/acronym

**DIVECCOT: D**iscovery of **I**deal **V**ariables and **E**xcellence markers in paediatric **R**apid **R**esponse **T**eams (DIVECCOT)

## Protocol version number and date: Protocol Version 1.0, 22^nd^ June 2022

## Research reference numbers

| **IRAS Number** | Not applicable |
| --- | --- |
| **Sponsor reference number** | **Not applicable** |
| **ISRCTN number** | Not applicable |
| **REC reference number** | Not applicable |

This protocol has regard for the HRA guidance

## Signature page

The undersigned confirm that the following protocol has been agreed and accepted and that the Chief Investigator agrees to adhere to the signed University of Birmingham’s Sponsorship CI declaration.

I agree to ensure that the confidential information contained in this document will not be used for any other purpose other than the evaluation or conduct of the investigation without the prior written consent of the Sponsor

I also confirm that I will make the findings of the study publicly available through publication or other dissemination tools without any unnecessary delay and that an honest accurate and transparent account of the study will be given; and that any discrepancies from the study as planned in this protocol will be explained.

Chief Investigator:

Signature: ...................................................................................................... Date: ......../……...../…….....

Name: (please print):......................................................................................................

## Sponsor statement:

Where the University of Birmingham takes on the sponsor role for protocol development oversight, the signing of the IRAS form by the sponsor will serve as confirmation of approval of this protocol.

##

## Table of Contents

[Full/long title of the study 1](#_Toc201688600)

[Short study title/acronym 1](#_Toc201688601)

[Protocol version number and date: Protocol Version 1.0, 22^nd^ June 2022 1](#_Toc201688602)

[Research reference numbers 1](#_Toc201688603)

[Signature page 1](#_Toc201688604)

[Sponsor statement: 1](#_Toc201688605)

[Table of Contents 2](#_Toc201688606)

[Key study contacts 3](#_Toc201688607)

[Study summary 3](#_Toc201688608)

[Funding and support in kind 4](#_Toc201688609)

[Role of study sponsor and funder 4](#_Toc201688610)

[Roles and responsibilities of study management committees/groups and individuals. 4](#_Toc201688611)

[Protocol contributors 4](#_Toc201688612)

[Key Terms and Abbreviations 5](#_Toc201688613)

[Study flow chart 6](#_Toc201688614)

[1. Study protocol 7](#_Toc201688615)

[2. Background 7](#_Toc201688616)

[3. Rationale 8](#_Toc201688617)

[4. Research question/aims 9](#_Toc201688618)

[What is the current provision and configuration of Critical Care Outreach Teams (CCOT) in paediatric tertiary centres across the UK and Republic of Ireland? 9](#_Toc201688619)

[4.1 Objectives 9](#_Toc201688620)

[1. To examine the current provision of Paediatric Critical Care Outreach Teams (PCCOT) in tertiary centres across the UK and Republic of Ireland. 9](#_Toc201688621)

[4.2 Outcomes 9](#_Toc201688622)

[5. Study design and methods of data collection and data analysis 9](#_Toc201688623)

[6. Study setting 11](#_Toc201688624)

[7. Participant recruitment 12](#_Toc201688625)

[7.1 Eligibility Criteria 12](#_Toc201688626)

[7.1.1 Inclusion criteria 12](#_Toc201688627)

[7.1.2 Exclusion criteria 12](#_Toc201688628)

[7.2 Recruitment target 12](#_Toc201688629)

[7.2.1 Size of recruitment target 12](#_Toc201688630)

[7.2.2 Recruitment technique 12](#_Toc201688631)

[7.3 Recruitment 12](#_Toc201688632)

[7.3.1 Participant identification 13](#_Toc201688633)

[7.3.2 Consent 13](#_Toc201688634)

[8. Safety reporting 13](#_Toc201688635)

[9. Ethical and regulatory considerations 13](#_Toc201688636)

[9.1 Assessment and management of risk 13](#_Toc201688637)

[9.2 Research Ethics Committee (REC) and other Regulatory review & reports 13](#_Toc201688638)

[Regulatory Review & Compliance 13](#_Toc201688639)

[Amendments 13](#_Toc201688640)

[9.3 Peer review 13](#_Toc201688641)

[9.4 Patient & Public Involvement 14](#_Toc201688642)

[9.5 Protocol compliance 14](#_Toc201688643)

[9.6 Data protection and patient confidentiality 14](#_Toc201688644)

[9.7 Indemnity 15](#_Toc201688645)

[9.8 End of study and archiving 15](#_Toc201688646)

[Access to the final study dataset 15](#_Toc201688647)

[10. 10.Dissemination policy 15](#_Toc201688648)

[10.1 Dissemination policy 15](#_Toc201688649)

[10.2 Authorship eligibility guidelines and any intended use of professional writers 16](#_Toc201688650)

[11. 11. References 16](#_Toc201688651)

[12. 12. Appendices 20](#_Toc201688652)

[12.1 Appendix 1- Required documentation 20](#_Toc201688653)

[12.2 Appendix 2 – the 43 step process for identification of deteriorating patient 21](#_Toc201688654)

[12.3 Appendix 3 – Schedule of Procedures 22](#_Toc201688655)

[12.4 Appendix 4 – Amendment History 23](#_Toc201688656)

## Key study contacts

| Student researcher | Bethan Holmes  Email: bethan.holmes@nhs.net |
| --- | --- |
| Chief Investigator | Dr Susan Neilson (Academic Supervisor)  Email: s.j.neilson@bham.ac.uk |
| Sponsor | **Not applicable** |
| Lead research site | Birmingham Women’s and Children’s Hospital National Health Service (NHS) Foundation Trust |

## Study summary

| Study Title | **D**iscovery of **I**deal **V**ariables and **E**xcellence markers in paediatric **R**apid **R**esponse **T**eams |
| --- | --- |
| Internal ref. no. (or short title) | DIVECCOT |
| Study Design | Explanatory sequential mixed method design |
| Study Participants | Professionals working within a Rapid Response Team in hospitals with a Paediatric Intensive Care Unit (PICU) in the United Kingdom and Republic of Ireland. |
| Planned Size of Sample (if applicable) | Phase one: CCOT member from 12-31 hospitals with a PICU |
| Follow up duration (if applicable) | N/A |
| Planned Study Period | 8 Weeks |
| Research Question/Aim(s) | To determine the provision and characteristics of PCCOTs in tertiary paediatric centres across the UK and Ireland |

## Funding and support in kind

| **FUNDER(S)** | **FINANCIAL AND NON FINANCIALSUPPORT GIVEN** |
| --- | --- |
| Health Education England (HEE)/National Institute of Health Research (NIHR) | Integrated Clinical Academic (ICA) Pre-doctoral Clinical Academic Fellow (PCAF) Programme – NIHR301010. |

## Role of study sponsor and funder

**This study does not have a sponsor.**

The funder requires a minimum of three days’ notice on publication of research outputs as per National Institute of Health Research (NIHR) Outputs and publication guidance (NIHR, 2021). The research outputs from this study will acknowledge all NIHR funding and support received for the research and include the NIHR disclaimer. Outputs may display the ‘Funded by NIHR’ logo, where appropriate,

## Roles and responsibilities of study management committees/groups and individuals.

There is no study management committee involvement in this study. The protocol contributors are the student’s clinical and academic supervisors within the Integrated Clinical Academic (ICA) Pre-doctoral Clinical Academic Fellow (PCAF) Programme.

There has been consultation with the local Patient and Public Involvement (PPI) group, the Young Person’s Advisory Group (YPAG) at Birmingham Women’s and Children’s (BWC) NHS Foundation Trust hospital in October 2021. They were presented oversight of whole study and felt that study covered an important topic area that was important for patient safety, parental reassurance and keeping patients out of Paediatric Intensive Care. Furthermore, there was consultation with YPAG in November 2021 regarding data collection tool development and changes were made according to the recommendations to ensure that the work remains patient and family focused and to help disseminate findings to the lay and professional audience

## Protocol contributors

Dr Heather Duncan (Birmingham Women’s and Children Hospital NHS Foundation Trust) (BWC)

Mrs Bethan Holmes (Student)

Dr Lucy Kelsall-Knight (University of Birmingham)

Dr Julie Menzies (Birmingham Women’s and Children Hospital NHS Foundation Trust) (BWC)

Dr Susan Neilson (University of Birmingham)

## Key Terms and Abbreviations

| Term | Description |
| --- | --- |
| Intensive Care Unit (ICU) | An ICU is a specially staffed and equipped area of a hospital dedicated to the monitoring and management of patients with life-threatening illnesses, injuries or complications. It provides expertise and facilities for support of vital functions and uses the skills of medical, nursing, and other personnel experienced in the management of these problems (Marshall et al, 2017)  Synonyms: Critical Care Services (CCS), Intensive Therapy Unit (ITU) |
| Early Warning Score (EWS) | EWS is a physiological score, which prescribes an appropriate response for the deteriorating patient in need of urgent medical care (Kolic et al, 2015). |
| Optimal components | The best or most favourable elements with the Rapid Response Team to create a successful larger whole.  For example, team member characteristics. |
| Quality | Degree of excellence (Marriam-Webster, 2020) |
| Quality metric | An objective measure of the quality of a process or service to allow quantification of the quality of a specific aspect of a process or service (Long, 2016).  Synonym: benchmark, standard, framework |
| Rapid Response System (RRS) | The structured approach to the management of the deteriorating patient outside of ICU which includes four components: the detection and escalation limb (involving EWS), the crisis response limb, the process improvement limb and the administrative limb (DeVita et al, 2006). |
| Critical Care Outreach Team (CCOT) | A group of professionals with an advanced skillset, additional to the ward-based team, that are activated on the identification of a deteriorating patient (Dacey et al, 2007).  Synonyms: Medical Emergency Team (MET), Rapid Response Team (RRT) and Patient at Risk Team (PAR) |

##

Study Flow chart

# Study protocol

# Background

This study will focus on the Critical Care Outreach Teams (CCOT) working within paediatric practice in the United Kingdom (UK) and Republic of Ireland (ROI). The CCOT are a group of professionals with an advanced skillset, additional to the ward-based team, that are activated on the identification of a deteriorating patient (Dacey et al, 2007). The CCOT forms the efferent, or response, limb of the Rapid Response System (RRS) which aims to mitigate preventable life threatening deterioration and death as well as reduce unplanned Intensive Care Unit (ICU) admissions (DeVita et al, 2006; Barocas et al, 2014)


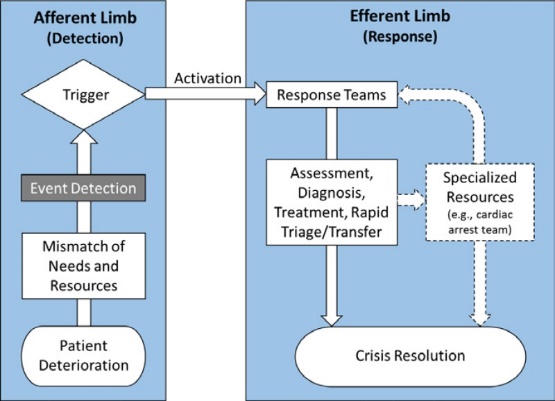


Figure 1 RRS (Hall et al, 2020)

The trigger for activating the CCOT for a deteriorating patient is nursing or family concern, or deviation from normal physiological parameters detected by a track and trigger tool such as the Early Warning Score (EWS) (Dean et al, 2008; Odel et al, 2009; Ray et al, 2009; Brady et al, 2015; Douw et al, 2015; Vorwerk and King, 2016; Lambert et al, 2017).

In clinical practice, when RRS identifies a deteriorating patient, the CCOT becomes involved at referral from a *“more senior clinician (who) hears the concerns and attends*” (Duncan, 2020: 2) and remains bedside until the patient is transferred to appropriate level of care. The stages within the RRS continuum is presented in its entirety in Appendix 2. In addition to rapidly responding to deteriorating patients on wards, the CCOT performs additional roles as shown in Table 2.

Table 2

|  | CCOT Role | - References |
| --- | --- | --- |
| 1 | Commencing initial management when called to a deteriorating patient and undertaking advanced clinical skills such as obtaining venous access and medication prescribing. | - Department of Health (DoH), (2000a), - DoH, (2001), - National Institute of Clinical Excellence (NICE) (2018), - Hogan et al (2019), - Hyde‐Wyatt and Garside (2020). |
| 2 | Avoiding unplanned Intensive Care Unit (ICU) admission or allowing ICU entrance in timely and safe manner. |  |
| 3 | Response to and management of medical and surgical emergencies requiring cardiopulmonary resuscitation. | - NICE (2018) |
| 4 | Patient review on ward following ICU discharge to prevent likelihood of ICU readmission and long term follow up post hospital discharge. | - DoH (2001), - Bellomo et al (2003; 2004), - Leary and Ridley (2003), - DeVita et al (2006), - Faculty of Intensive Care Medicine (FICM) (2015), - Hogan et al (2019). |
| 5 | Pastoral care: emotional, educational and practical support for ward nurses specifically those inexperienced in caring for critically ill patients in addition to collaboration and communication for patients and significant others. | - DoH (2000b), - DoH (2001), - Intensive Care Society (ICS) (2002), - FICM (2015), - Hyde‐Wyatt and Garside (2020). |
| 6 | Discussion regarding treatment limitation and ‘Do not attempt cardiopulmonary resuscitation’ (DNACPR) decisions | - Pederby et al (2007), - Chen et al (2008) - Barnard-Smith et al (2016) - Hyde‐Wyatt and Garside (2020). |
| 7 | Patient safety, clinical governance, audit and evaluation | - DoH (2000), - DoH (2001), - FICM (2015). |

Research reports wide variability between CCOT in different hospitals in terms of service delivery resources, type and availability of CCOT provision, quality of service provision and team membership characteristics including profession, level of education, skillset and clinical experience of team members (Esmonde et al, 2006; Chen et al, 2012; Cheng and Mikrogianakis, 2018; Lambert et al, 2017; NICE, 2018; Subbe et al, 2019). Wide variability can impact quality of care and increase expense therefore measurement of healthcare services using clinical benchmarks, a systematic process whereby current practice and care are compared to best practice and care, is advisable to ensure quality care delivery and cost effectiveness (DoH, 2001; DoH, 2010; Alderwick et al, 2017; RCN, 2017; Robertson, 2017). In recent years, clinical benchmarks, or quality metrics, have been developed that are specific to the RRS (National Outreach Forum (NOrF), 2020: Subbe et al, 2019) . Currently, these have not been used to compare current practice and care in CCOT’s in paediatric tertiary centres in the UK and Republic of Ireland (ROI). Furthermore, the optimum components within the CCOT have not well defined due to level of variability.

# Rationale

Within the UK, the CCOT exists in 80 to 85 percent of adult hospitals (NICE, 2018; Hogan et al, 2019) but in only 52 percent of paediatric tertiary centres (Roland et al, 2014). However, this research was seven years ago and as UK policy supports the development of CCOT, is likely unrepresentative of the current provision. Furthermore, as Roland et al (2014) did not focus primarily on the paediatric CCOT service, the attributes and characteristics of the existing paediatric CCOT team remains undetermined . Research undertaken in the UK to date has not primarily focused on or included all UK CCOTs in paediatric tertiary hospitals (Haines, 2005; Roland et al, 2014). Therefore, the data this study will add to current clinical and research knowledge is novel and will address the identified dearth of knowledge around ideal CCOT team structure, composition and configuration and the recognition of objective quality metrics (Esmonde et al, 2006; McGaughey et al, 2007; Salvatierra et al, 2014; Smith et al, 2017; Hamsen et al, 2018).

The research design for this study is a quantitative study, gathering information through a questionnsire. Though, McGaughey et al (2007) recommend that a multi-site Randomised Control Trial (RCT) would be a preferable design to allow for direct comparison between a centre with an CCOT and one without, it is not feasible as it is currently unknown where paediatric CCOTs are located in the UK and ROI. However, this study will generate provision data to inform a RCT study that may occur in the future.

Key concepts

- The CCOT aims to reduce undetected deterioration in patients in hospital alongside other functions,
- The provision, characteristic and governance of CCOTs in paediatric tertiary centres in the UK and ROI is unknown,
- Variability is common in CCOT and may influence the quality and cost effectiveness of the service.

# Research question/aims

What is the current provision and configuration of Critical Care Outreach Teams (CCOT) in paediatric tertiary centres across the UK and Republic of Ireland?

- 1. Objectives

1. To examine the current provision of Paediatric Critical Care Outreach Teams (PCCOT) in tertiary centres across the UK and Republic of Ireland,
2. To identify and describe the key characteristics of PCCOTs, including team composition, operational models, and service delivery practices,
3. To explore the governance structures of PCCOTs and the collection and use of quality metrics to assess team performance and patient outcomes.

4.2 Outcomes

This study will provide enhanced knowledge of paediatric PCCOT service provision in the UK and ROI by examining current provision, workforce characteristics and governance structures.

# Study design and methods of data collection and data analysis

Study design

Descriptive cross-sectional design

Pre-data collection

1. A scoping review has been undertaken to establish the team characteristics required to assemble the best CCOT “*work-as-imagined*” (Hollnagal et al, 2015: 18) and the appropriate metrics to measure quality of service delivery as identified in current research literature.
2. The protocol and study approach were presented to the local Patient and Public Involvement (PPI) group at Birmingham Women’s and Children’s Hospital NHS Foundation Trust (BWCH), the Young Persons Advisory Group (YPAG) in October 2021. The YPAG expressed positive views on the study and felt it was valuable as looked at an unexplored area of research and expressed interest in reviewing the data collection tools once prepared.
3. Peer review of data collection tools as described in next section.

Data Collection Methods

Please note, for the duration of this protocol the student researcher, Bethan Holmes, will be referred to as the researcher.

Data collection tool development: questionnaire

1. Content of questionnaire was informed by scoping review findings.
2. Review process for questionnaire:
   1. Review by mentor team,
   2. PPI review: The YPAG at BWCH were consulted about the questionnaire formatting to determine important elements from young person’s and potential patient group perspective. Alterations were made as suggested,
   3. Review by a local expert in qualitative research design,
   4. Review by international expert in quantitative methods and questionnaire design,
   5. The questionnaire will be transferred onto the online survey platform Bristol Online Survey (BOS). A further review by two members of BWC PCCOT will occur once the questionnaire is online to assess the ability to undertake questionnaire on different devices including a mobile phone. Phase one Procedures
3. **Week 1**:
   1. Study recruitment commences: a recruitment flyer (see appendix 1) containing the researcher’s email address will be distributed through professional special interest groups and social media including but not limited to:

- PCCS,
- National Outreach Forum (NOrF),
- The researcher’s personal twitter account.
  1. Interested individuals will express interest in the study by contacting the researcher via email. The researcher will reply with an encrypted email containing Participant Information Sheet (PIS-Questionnaire), informed consent form (ICF-questionnaire) and instructions regarding the arrangements for discussion and signing of the consent form via teleconferencing platform. Participant will be given a minimum of 24 hours to consider taking part in the study. After this period, a video conferencing platform call will be arranged between the researcher and participant. Within this call, the participant will be given the opportunity to ask further questions and sign the ICF-questionnaire as witnessed by the researcher.
  2. The participant will return the completed ICF-questionnaire to the researcher, the researcher will complete the witness section on the ICF-questionnaire and a link to the study questionnaire on the online survey platform BOS will be forwarded to the participant.
  3. If the participant prefers, paper copies of the PIS-questionnaire, ICF-questionnaire and questionnaire can be posted to the participant.

1. **Week 3**: Repeat actions a to c as performed in week 1. The researcher will extend the questionnaire opening by two weeks.
2. **Week 5:** Repeat actions a to c as performed in week 1. The researcher will extend the questionnaire opening by two weeks.
3. **Week 7:** Questionnaire now closed. Analysis of quantitative data will begin.
4. -responders from email communication at week 7 and document communication on screening log. Researcher will consider if needs to re-recruit participants who did not fit initial screening criteria for phase 2.

Data analysis methods

1. Phase one

- The researcher will enter the phase one data onto a spread sheet within Microsoft Excel. Column 1 will contain the participant anonymised unique identifier. Column 2-40 will contain the participants’ responses in the questionnaire.
- The answers to each question will be assigned a numerical code,
- Accuracy of the transfer of the data from the questionnaire to the spreadsheet and the coding will be verified by a member of the supervisory team with experience of quantitative research analysis.
- From the extracted data, descriptive statistical analysis will be undertaken; measures of central tendency and variability for continuous variables will be computed and verified by supervisory team.

# Study setting

This is a multi-centre study which includes the 29 hospitals with a Paediatric Intensive Care Unit (PICU) in the UK and ROI as identified by Paediatric Intensive Care Audit Network (PICAnet, 2021) as listed in Figure 3.

| Figure 3: UK and ROI hospitals with a PICU (PICAnet, 2021) | |
| --- | --- |
| Addenbrooke's Hospital, Cambridge | Leicester Royal Infirmary |
| Alder Hey Children’s Hospital, Liverpool | Noah’s Ark Children’s Hospital for Wales, Cardiff |
| Birmingham Children’s Hospital | Nottingham Children’s Hospital |
| Bristol Royal Hospital for Children | Royal Belfast Hospital for Sick Children |
| Children’s Health Ireland at Crumlin, Dublin | Royal Brompton Hospital, London |
| Children’s Health Ireland at Temple Street, Dublin | Royal Hospital for Children, Glasgow |
| Evelina London Children’s Hospital | Royal Hospital for Sick Children, Edinburgh |
| Freeman Hospital, Newcastle upon Tyne | Royal Manchester Children’s Hospital |
| Glenfield Hospital, Leicester | Royal Stoke University Hospital |
| Great Ormond Street Hospital, London (PICU/NICU/CICU) | Sheffield Children’s Hospital |
| Great North Children’s Hospital, Newcastle upon Tyne | Southampton Children’s Hospital |
| James Cook University Hospital, Middlesbrough | St Mary’s Hospital, London |
| John Radcliffe Hospital, Oxford | St George’s Hospital, London |
| King’s College Hospital, London | The Royal London Hospital |
| Leeds General Infirmary |  |
|  |  |

All data collection will occur online via electronic questionnaire/questionnaire and interview via video conferencing platform, so there are no specific site requirements or differential activities on various sites.

# Participant recruitment

## Eligibility Criteria

7.1.1 Inclusion criteria

Phase one

- Over age of 18 years old
- Healthcare professional working within a tertiary NHS hospital with a PICU in UK or Ireland (n=29)
- Informed Consent Form completed.

7.1.2 Exclusion criteria

Phase one

- Hospitals with a PICU without an CCOT that answered no to the screening question,
- Adult CCOT services in the UK and ROI that review adults only,
- Any CCOT service outside the UK and ROI.

## Recruitment target

7.2.1 Size of recruitment target

Phase one

**Size:** completed questionnaires from 12-29 paediatric tertiary centres

**Rationale:** There are currently 29 NHS hospitals with a PICU in the UK and ROI (PICaNet, 2021). In 2014, CCOT were present in 52 percent of these hospitals (Roland et al, 2014); however, the current value is an unknown. It is acknowledged that questionnaire participation rate can be as low as 40 percent (Nayak and Narayan, 2019) hence the low minimum sample target (n=12). There has been thoughtful consideration regarding the data collection tool and despite the potential for low participant rate, it is felt by the student and the supervisory team that an online questionnaire is the best method to collect the quantitative data remotely.

7.2.2 Recruitment technique

Phase one: stratified purposive sampling

7.3 Recruitment

Recruitment to phase one

1. The population of interest are professionals working within paediatric CCOT in UK or ROI. Potential participants will be self-identifying; after seeing a study flyer circulated by the professional interest groups, the potential participant will contact the researcher via email.
2. The researcher will then provide the potential participant with PIS-questionnaire, ICF-questionnaire and opportunity for discussion about the study and the consent process. The consent form will be signed whilst on call to researcher to allow witnessing of signature.
3. It is recognised that CCOT professionals who missed the first circulation of the study flyer may like the opportunity to participate in the study, hence the flyer will be circulated by the professional interest groups and social media on a further two occasions (a total of three times).

7.3.1 Participant identification

- Participants will be recruited publicly from professional special interest groups (PCCS and NOrF) and social media using a flyer containing the researcher’s contact details.
- Participants will be self-identifying as they will approach the researcher using these contact details.
- Participants will receive no payment.

7.3.2 Consent

The participant will email the researcher to express interest in the study. The researcher will email the participant two documents: PIS-questionnaire and ICF-questionnaire. The participant will read the information on PIS-questionnaire and have opportunity for thorough and interactive communication with the researcher to ensure the potential participant understands what is involved in the study. The participant will then complete ICF-questionnaire whilst on a video conferencing platform call to the researcher to ensure the signature is witnessed. As this is a low risk study, a simple electronic, such as typed name or a stylus or finger drawn signature, is adequate when seeking consent (Health Research Authority, 2018). The participant will be asked to screenshare on the video conferencing platform whilst they sign ICF-questionnaire if they chose to sign electronically. The consent form will then be returned to the researcher via email.

# Safety reporting

Not applicable to this research study.

# Ethical and regulatory considerations

## 9.1 Assessment and management of risk

Due to the subject nature and the population, this study is unlikely to cause potential risk or harm or encounter safeguarding issues.

## 9.2 Research Ethics Committee (REC) and other Regulatory review & reports

**This study has been received ethical review at University of Birmingham.**

## Regulatory Review & Compliance

- Prior to participant enrolment, the researcher will ensure that appropriate approvals from participating organisations are in place.
- For any amendment to the study, the researcher in agreement will submit information to the appropriate body for them to issue approval for the amendment.

## Amendments

- If the researcher feels an amendment is required, she will discuss with the supervisory team to decide whether this is a substantial amendment and will discuss with the University of Birmingham Ethics team.
- Any amendment will be documented to ensure tracking of the most recent protocol version.

## 9.3 Peer review

- This study has received independent peer review as part of the funding process for NIHR Pre-doctoral Clinical Academic Fellowship (PCAF).
- Protocol and associated documentation (both consent form, both PIS, questionnaire and interview topic guide) developed with support from supervisory team with expertise in clinical care and research design.
- The questionnaire has received the following peer review:

1. Review by a local expert in qualitative research design,
2. Review by international expert in quantitative methods and questionnaire design,
3. Questionnaire pilot by four members of BWCH RTT to determine appropriate phraseology, terminology, style and understandability and changes made as required. These team members will not undertake the questionnaire in the study,
4. The questionnaire will be transferred onto the online survey platform Bristol Online Survey (BOS). A further review by two members of BWCH RTT will occur once the questionnaire is online to assess the ability to undertake questionnaire on different devices including a mobile phone.

## 9.4 Patient & Public Involvement

There has been consultation with the local Patient and Public Involvement (PPI) group, the Young Person’s Advisory Group (YPAG) at BWCH:

In October 2021, the researcher presented oversight of whole study to the YPAG who collectively felt that study covered an important topic area which was significant for patient safety, parental reassurance and keeping patients out of Paediatric Intensive Care.

In November 2021, the researcher consulted with YPAG regarding data collection tool development and altered both the questionnaire and interview schedule on the feedback to ensure that the work remains patient and family focused.

Once the study is complete, the researcher will return to the YPAG to feedback the result of the study.

## 9.5 Protocol compliance

Accidental protocol deviations can occur from time to time, these will be documented on the relevant paperwork. Deviations deemed to be reportable such as protocol breach, breach of confidentiality, or breach of Good Clinical Practice (GCP) that could affect the integrity of the data will be reported to the appropriate persons as soon as the researcher is aware.

## 9.6 Data protection and patient confidentiality

Data will be collected and stored in accordance with the Data Protection Act 2018 and General Data Protection Regulations.

The data will be generated by this study is as follows**:**

Phase one

- Consent form (for personal detail storage)
- Survey results
- Quantitative data analysis data such as descriptive statistics

Analysis and summary of research data

- Final research report

Data Storage

- Anonymised data from the study will be stored on secure University of Birmingham research data store. Any sensitive data on local or removable drives will be encrypted.
- Paper copies of consent forms and completed questionnaires (if participants decide on this option) will be stored in the site file in a code protected locker.
- Personally identifiable data, such as the hospital the participant works at, is needed in this study. However, the only person to see the data its raw form will be the researcher and she will act as the data custodian:
- Raw data files will be allocated a participant reference number before uploading and change any names (people and places) in the text to pseudonyms,
- Data will not be transferred or transmitted to co-investigators with personally identifiable information available as the information will be coded as discussed above. The required data will be stored securely in separate locations using encrypted digital passwords.
- On a weekly basis, the researcher will upload anonymised research data into the University of Birmingham Research Data Store (RDS). Backup copies of data are taken daily, and data is stored in separate buildings from the live data. Each place where data is stored will be given a reference number to ensure accurate but secure record keeping

## 9.7 Indemnity

NHS indemnity scheme will apply as protocol author has an NHS contract.

## 9.8 End of study and archiving

According to the University of Birmingham data retention guidance, the following data storage will occur for the data collected in this study;

- Personal data will not be stored for longer than is necessary,
- At the production of the final research report, a subset of the data that underpins the report such as the anonymised questionnaire data, consent forms and transcribed interviews will be transferred to the University of Birmingham Research Data Archive (RDA). Once transferred the data will be set to read-only to prevent any inadvertent additions or deletions of the dataset,
- Any changes will result in a new dataset, which will be archived separately. The RDA solution has been created to be highly resilient and is located at two data centres in two different sites, with a backup placed in a third site*.*
- Anonymised data will be stored for 10 years, should access to the data be requested within a 10 year period, the 10 year clock is then reset from the point of last access. After the 10 year period the data will be deleted.
- The arrangements for disposal of data will adhere to University of Birmingham Research Data Management policy.

# Dissemination policy

## Dissemination policy

1. On completing of the study, the data will be analysed and tabulated, and Final Study Report prepared,
2. The Final Study Report will form a thesis for the researchers’ master’s degree in clinical health research at University of Birmingham,
3. The researcher plans to disseminate the data obtained within this study including full study report, anonymised participant level dataset and statistical code through publication and attendance at national and international conferences. This will be concluded within one year of study completion.
4. The participating investigators have right to publish anonymised study data and will also assist in study findings dissemination,
5. The Funding body (NIHR) will be notified and acknowledged within the publications and/or conference posters,
6. During the quantitative phase, participants will be given the opportunity to share their contact details to receive feedback of findings after study completion. Once the Final Study Report is completed, this participant group will be emailed and invited to attend a presentation on the study outcomes. They will also be given access to the Final Study Report and publications, if requested.
7. The researcher will feedback to the local CCOT who assisted in the piloting of the questionnaire,
8. The researcher will feedback study findings to BWCH YPAG who assisted in development of study designs and data collection tool

## 10.2 Authorship eligibility guidelines and any intended use of professional writers

The researcher and the supervisory team will be acknowledged as authors in accordance with defined authorship guidance.

# References

Alderwick, H., Charles, A., Jones, B. and Warburton, W. (2017) *Making the care for quality improvement: lessons for NHS boards and leaders*. Available at: https://www.kingsfund.org.uk/publications/making-case-quality-improvement (Accessed: 15 March 2021)

Bailey, J. (2008) ‘First Steps in Qualitative Data Analysis: Transcribing.’ *Family Practice* 25 (2): 127-131

Banard-Smith, J., Lighthall, G.K., Subbe, C.P., Durham, L., Welch, J., Bellomo, R. and Jones, D.A., (2016) ‘Clinical outcomes of patients seen by rapid response teams: a template for benchmarking international teams’, *Resuscitation*, 107, pp.7-12.

Barocas, D.A., Kulahalli, C.S., Ehrenfeld, J.M., Kapu, A.N., Penson, D.F., You, C.C., Weavind, L. and Dmochowski, R. (2014) ‘Benchmarking the use of a rapid response team by surgical services at a tertiary care hospital’, *J Am Coll Surg*, 218 (1), pp. 66-72.

Bellomo, R., Goldsmith, D., Uchino, S., Buckmaster, J., Hart, G., Opdam, H., Silvester, W., Doolan, L. and Gutteridge, G. (2004), ‘Prospective controlled trial of effect of medical emergency team on postoperative morbidity and mortality rates’, *Critical care medicine*, *32* (4), pp.916-921.

Brady, P.W., Zix, J., Brilli, R., Wheeler, D.S., Griffith, K., Giaccone, M.J., Dressman, K., Kotagal, U., Muething, S. and Tegtmeyer, K. (2015) ‘Developing and evaluating the success of a family activated medical emergency team: a quality improvement report’, *BMJ Quality and Safety*, 24, pp. 203-211.

Chen, J., Flabouris, A., Bellomo, R., Hillman, K. and Finfer, S. (2008), ‘The Medical Emergency Team System and not-for-resuscitation orders: results from the MERIT study’, *Resuscitation*, *79* (3), pp.391-397.

Chen, J.G., Kemper, A.R., Odetola, F., Cheifetz, I.M. and Turner, D.A. (2012), ‘Prevalence, characteristics, and opinions of pediatric rapid response teams in the United States’, *Hospital Pediatrics*, *2* (3), pp.133-140.

Cheng, A. and Mikrogianakis, A. (2018) ‘Rapid Response System for paediatrics: Suggestions for optimal organisation and training’, *Pediatrics & Child Health*, 23 (1): pp. 51–57.

Creswell, J. W. and Plano Clark, V. L. (2018). *Designing and conducting mixed methods research* (3rd ed.). Thousand Oaks, CA: Sage.

De Allegri, M., Brenner, S., Kambala, C., Mazalale, J., Muula, A.S., Chinkhumba, j., Wilhelm, D. and Lohmann, J. (2020) ‘Exploiting the emergent nature of mixed methods designs: insights from a mixed methods impact Evaluation in Malawi’, *Health Policy and Planning*, 35 (1), pp. 102–106.

Dean, B.S., Decker, M.J., Hupp, D. Urbach, A.H., Lewis, E., and Benes-Stickle, J. (2008) ‘Condition HELP: A Pediatric Rapid Response Team Triggered by Patients and Parents’, *Journal for Healthcare Quality*, 30 (3), pp. 28-31.

Department of Health (DoH, 2000a) *Comprehensive Critical Care: a review of adult critical care services*. Available at: https://webarchive.nationalarchives.gov.uk/20090608051759/http://www.dh.gov.uk/en/Publicationsandstatistics/Publications/PublicationsPolicyAndGuidance/DH_4006585 (Accessed: 7 July 2021)

Department of Health (DoH, 2000b) *Liberating the NHS: Developing the Healthcare Workforce*. Available at: <https://assets.publishing.service.gov.uk/government/uploads/system/uploads/attachment_data/file/216421/dh_132087.pdf> (Accessed: 4 August 2021)

Department of Health (DoH) (2001) *The Nursing Contribution to the provision of Comprehensive Critical Care for Adults: a strategic program of action*. Available at: http://anaesthesiaconference.kiev.ua/downloads/nursing%20contribution%20to%20comprehensive%20critical%20care_2005.pdf (Accessed: 6 August 2021)

Department of Health (2010) *Essence of Care.* Available on: <https://www.gov.uk/government/publications/essence-of-care-2010> (Accessed: 13 March 2021)

DeVita, M.A., Bellomo, R., Hillman, K.A., Rotondi, A., Teres, D., Auerbach, A., Chen, W.J., Duncan, K.D., Kenward, G., Bell, M., Buist, M., Chen, J., Bion, J.F., Kirby, A., Lighthall, G.K., Ovreveit, J., Braithwaite, S., Gosbee, J., Milbrant, E., Peberdy, M.A., Savitz, L., Young, L. & Galhotra, S. (2006) ‘Findings of the first consensus conference on medical emergency teams’, *Crit Care Med*, 34 (9), pp. 2463-2478.

Douw, G., Schoonhoven, L., Holwerda, T., Huisman-de Waal, G., Van Zanten, A. R., Van Achterberg, T., and Van der Hoeven, J.G. (2015) ‘Nurses' worry or concern and early recognition of deteriorating patients on general wards in acute care hospitals: a systematic review’, *Critical care*, *19,* 230 (11pp). doi:10.1186/s13054-015-0950-5.

Duncan, H. and Hudson, A.P (2020) ‘Implementation of a paediatric early warning system as a complex health technology intervention’, *Archives of Disease in Childhood*, *106* (3), pp.215-218.

Esmonde, L., McDonnell, A., Ball, C., Waskett, C., Morgan, R., Rashidian, A., Bray, K., Adam, S. and Harvey, S. (2006), ‘Investigating the effectiveness of critical care outreach services: a systematic review’, *Intensive care medicine*, *32* (11), pp.1713-1721.

Foster, E.M., Fang, G.Y. and Conduct Problems Research Group (2004), ‘Alternative methods for handling attrition: an illustration using data from the Fast Track evaluation’, *Eval Rev*. 28 (5), pp. 434-464.

Graneheim, U. and Lundman, B. (2004) ‘Qualitative Content Analysis in Nursing Research: Concepts, Procedures and Measures to Achieve Trustworthiness’, *Nurse Education Today* 24, pp. 105-112

Guetterman, T.C., Fetters, M.D. and Creswell, J.W. (2015), ‘Integrating quantitative and qualitative results in health science mixed methods research through joint displays’, *The Annals of Family Medicine*, 13 (6), pp. 554-561.

Hall K.K., Shoemaker-Hunt, S., and Hoffman, L. (2020) *Making Healthcare Safer III: A Critical Analysis of Existing and Emerging Patient Safety Practices*. Rockville (MD): Agency for Healthcare Research and Quality (US), illus. Available at: https://www.ncbi.nlm.nih.gov/books/NBK555513/figure/ch5.fig1/ (Accessed 09 July 2021)

Haines, C. (2005) ‘Acutely ill children within ward areas – care provision and possible development strategies’, *Nursing in Critical Care*, 10 (2), pp. 98-104

Health Research Authority (HRA) (2021) *UK Policy Framework for Health and Social Care Research* Available on: (Accessed: 4 April 2022)

Health Research Authority (2018) Joint Statement on seeking consent by electronic methods Available on: https://s3.eu-west-2.amazonaws.com/www.hra.nhs.uk/media/documents/hra-mhra-econsent-statement-sept-18.pdf (Accessed: 11 March 2022)

Hogan, H., Hutchings, A., Wulff, J., Carver, C., Holdsworth, E., Welch, J., Harrison, D. and Black, N., (2019). ‘Interventions to reduce mortality from in-hospital cardiac arrest: a mixed-methods study’, *Health Services and Delivery Research*, *7* (2), pp.1-110.

Hollnagel, E., Wears, R.L. and Braithwaite J. (2015) *From Safety-I to Safety-II: A White Paper.* Available at: https://www.england.nhs.uk/signuptosafety/wp-content/uploads/sites/16/2015/10/safety-1-safety-2-whte-papr.pdf (Accessed: 6 August 2021)

Hyde‐Wyatt, J. and Garside, J. (2020) ‘Critical care outreach: A valuable resource?’ *Nursing in Critical Care*, *25* (1), pp.16-23.

Intensive Care Society (ICS) (2002) *Guidelines for the Introduction of Outreach Services* Available on: http://anaesthesiaconference.kiev.ua/downloads/ICU_standards-outreach_2002.pdf (Accessed 4^th^ August 2021)

Ivankova. N.V. and Kawamura, Y. (2010) ‘Emerging trends in the utilization of integrated designs in the social, behavioural, and health sciences’ in Tashakkori, A. and Teddlie, C. (ed.) *Sage Handbook of Mixed Methods in Social and Behavioral Research*. California: Sage Publications, pp. 581-611.

Kolic, I., Crane, S., McCartney, S., Perkins, Z. and Taylor, A., 2015. Factors affecting response to national early warning score (NEWS). *Resuscitation*, *90*, pp.85-90.

Lambert, V., Matthews, A., MacDonnell, R. & Fitzsimons, S. (2017) ‘Paediatric early warning systems for detecting and responding to clinical deterioration in children: a systematic review’, *BMJ Open*, 7, 014497 (13pp). doi:10.1136/bmjopen-2016- 014497

Leary, T. and Ridley, S. (2003) ‘Impact of an outreach team on re‐admissions to a critical care unit’, *Anaesthesia*, *58* (4), pp.328-332.

Long, M. (2016) *Quality Metrics: what does it really mean?* Available at: https://www.contractpharma.com/issues/2016-03-01/view_features/quality-metrics-what-does-it-really-mean (Accessed: 11 October 2021).

Marriam-Webster (2020) *Quality* Available at:

https://www.merriam-webster.com/dictionary/quality?src=search-dict-box (Accessed: 11 October 2021)

McGaughey, J., Alderdice, F., Fowler, R., Kapila, A., Mayhew, A. and Moutray, M. (2007) ‘Outreach and Early Warning Systems (EWS) for the prevention of intensive care admission and death of critically ill adult patients on general hospital wards’, *Cochrane Database of Systematic Reviews*, (3).

National Institute for Health and Care Excellence (NICE) (2018) *Chapter 27 Critical Care Outreach Teams: Emergency and Acute Medical Care in over 16’s: service delivery and organisation: NICE Guideline 94.* Available at: https://www.nice.org.uk/guidance/ng94/evidence/27.critical-care-outreach-teams-pdf-172397464640 (Accessed: 14 July 2021)

National Institute of Health Research (NIHR) (2021) *NIHR research outputs and publication guidance* Available at: <https://www.nihr.ac.uk/documents/nihr-research-outputs-and-publications-guidance/12250> (Accessed: 4 April 2022)

National Outreach Forum (NOrF) (2020) *Quality and Operational Standards for the Provision of Critical Care Outreach Services: National Outreach Forum*. Available at: https://www.norf.org.uk/resources/Documents/QOS%20CCOS%20NOrF/NOrF%20QOS%20Final%20December%202020.pdf (Accessed: 3 March 2021).

Nayak, M.S.D.P. and Narayan,K.A. (2019) ‘Strengths and weaknesses of online surveys’, *IOSR Journal of Humanities and Social Sciences,* 24 (5), pp. 31-38.

Marshall, J.C., Bosco, L., Adhikari, N.K., Connolly, B., Diaz, J.V., Dorman, T., Fowler, R.A., Meyfroidt, G., Nakagawa, S., Pelosi, P. and Vincent, J.L. (2017) ‘What is an intensive care unit? A report of the task force of the World Federation of Societies of Intensive and Critical Care Medicine’ *Journal of critical care*, *37*, pp.270-276.

Obwuegbuzie, A.J. and Collins, K.M.T (2007) ‘A Typology of Mixed Methods Sampling Designs in Social Science Research’, *The Qualitative Report*, 12 (2), pp. 281-316

Onwuegbuzie, A. J., Teddlie, C. (2003). A framework for analyzing data in mixed methods research. In Tashakkori, A., Teddlie, C. (Eds.), SAGE handbook of mixed methods in social & behavioral research (pp. 351-383). Thousand Oaks, CA: Sage.

Odell, M., Victor, C. and Oliver, D. (2009) ‘Nurses’ role in detecting deterioration in ward patients: Systematic literature review’, *J. Adv. Nurs.* *65*, pp. 1992–2006.

Pederdy, M.A., Cretikos, M., Abella, B.S., DeVita, M., Goldhill, D., Kloeck, W., Kronick, S.L., Morrison, L.J., Nadkarni, V.M., Nichol, G. and Nolan, J.P. (2007), ‘Recommended guidelines for monitoring, reporting, and conducting research on medical emergency team, outreach, and rapid response systems: an Utstein-style scientific statement: a scientific statement from the International Liaison Committee on Resuscitation’, *Circulation*, *116* (21), pp.2481-2500.

Paediatric Intensive Care Audit Network (PICANet) (2021) *Annual Report 2021* Available at: https://www.picanet.org.uk/wpcontent/uploads/sites/25/2021/02/PICANet2020_AnnualReportSummary_v1.0.pdf (Accessed 27 September 2021)

Plano Clark, V.L. (2010) The adoption and practice of mixed methods: U.S. trends in federally funded health-related research. *Qual Inq*., 16 (6): 428-440.

Ray, E.M., Smith, R., Massie, S., Erickson, J., Hanson, C., Harris, B. and Willis, T.S. (2009) ‘Family alert: implementing direct family activation of a pediatric rapid response team’, *Jt Comm J Qual Patient Saf*, 35 (11), pp. 575-80.

Robertson, R. (2017) *How does the NHS compare internationally? Big election questions.* Available at: <https://www.kingsfund.org.uk/publications/articles/big-election-questions-nhs-international-comparisons> (Accessed: 13 March 2021)

Roland, D., Oliver, A., Edwards, E.D.*,* Mason, B.W. and Powell, C.V.E. (2014)

‘Use of paediatric early warning systems in Great Britain: has there been a change of practice in the last 7 years?’ *Archives of Disease in Childhood,*99**:** 26-29.

Royal College of Nursing (RCN) (2017) *Understanding Benchmarking* Available from: <https://www.rcn.org.uk/professional-development/publications/pub-006333> (Accessed: 13 March 2021)

Salvatierra, G., Bindler, R.C., Corbett, C., Roll, J. and Daratha, K.B. (2014), ‘Rapid response team implementation and in-hospital mortality’, *Critical care medicine*, *42* (9), pp.2001-2006.

Schoonenboom, J., and Johnson, R. B. (2017), ‘How to Construct a Mixed Methods Research Design’, *Kolner Zeitschrift fur Soziologie und Sozialpsychologie*, *69* (Suppl 2), pp. 107–131.

Shenton, A.K. (2004), ‘Strategies for ensuring trustworthiness in qualitative research projects’,  *Education for information*, *22*(2), pp.63-75.

Smith, P.L. and McSweeney, J. (2017), ‘Organizational perspectives on rapid response team structure, function, and cost: a qualitative study’, *Dimensions of Critical Care Nursing*, *36* (1), pp.3-13.

Subbe, C.P., Bannard-Smith, J., Bunch, J., Champunot, R., DeVita, M.A., Durham, L., Edelson, D.P., Gonzalez, I., Hancock, C., Haniffa, R., Hartin, J., Haskell, H., Hogan, H., Jones, D.A., Kalkman, C.J., Lighthall, G.K., Malycha, J., Ni, M.Z., Phillips, A.V., Robulotta, F., So, R.K. and Welch, J. (2019) ‘Quality Metrics for the evaluations of Rapid Response Systems: Proceedings from the third international consensus conference on Rapid Response Systems’, *Resuscitation*, 141, pp. 1-12.

The Faculty of Intensive Care Medicine (FICM) (2015) *Guidelines for the Provision of Intensive Care Services*. Available on: https://www.ficm.ac.uk/sites/default/files/GPICS%20-%20Ed.1%20%282015%29_0.pdf (Accessed: 14 July 2021)

Vorwerk, J. and King, L. (2016) ‘Consumer participation in early detection of the deteriorating patient and call activation to rapid response systems: a literature review’, *Journal of Clinical Nursing*, 25 (1-2), pp. 38–42

# 12. Appendices

## 12.1 Appendix 1- Required documentation

- Recruitment leaflet
- Participant Informed Consent Form (ICF) - Questionnaire,
- Participant Information Sheet (PIS) – Questionnaire


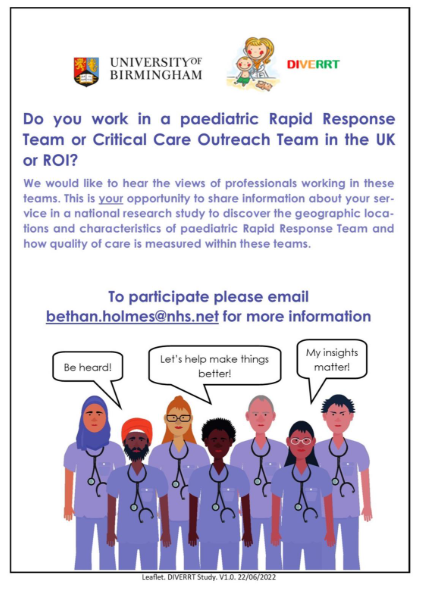


## 12.2 Appendix 2 – the 43 step process for identification of deteriorating patient

*A patient is observed measuring appropriate vital signs (1) accurately (2), frequently enough (3) so the trend of deterioration (4) is visible (5) and recognised (6). The nurse interprets the condition as deterioration (7), initiates a treatment plan (8) calls a doctor/more senior clinician (9) to communicate their concern (10) effectively (11) and request for attendance (13). The doctor/more senior clinician hears the concerns (14) and attends (15) in a timely way (16) to appropriately assess the patient (17), interpret the deterioration information (18) update the management plan (19) with the nurse (20) and the patient/parent (21). They call a more senior doctor/clinician (22) with critical care skills (23) and communicate their concerns (24) effectively and the treatment effect of initial management (26) with a request (27) for attendance (28) within the appropriate time period (29). The critical care clinician attends (30), assesses the patient (31) and the treatment effect (32) in a timely (33) and appropriate manner (34). The management plan is updated (35) with the bedside team (36), the patient’s own senior clinician (37) and the patient/parent (38). The patient is monitored (39) and stabilised (40) prior to transfer (41) to an appropriate critical care area (42) where care is effectively handed over (43)”*

(Duncan, 2020: 2

## 12.3 Appendix 3 – Schedule of Procedures

|  | Week Number | | | | | | | |
| --- | --- | --- | --- | --- | --- | --- | --- | --- |
| **Procedures** | 1 | 2 | 3 | 4 | 5 | 6 | 7 |  |
| Distribution of recruitment flyer. PIS, IFC and questionnaire to potential participants. |  |  |  |  |  |  |  |  |
| Procedures as week 1 |  |  |  |  |  |  |  |  |
| Procedures as week 1 |  |  |  |  |  |  |  |  |
| Close questionnaire |  |  |  |  |  |  |  |  |
| Analysis |  |  |  |  |  |  |  |  |

##

## 12.4 Appendix 4 – Amendment History

| **Amendment No.** | **Protocol version no.** | **Date issued** | **Author(s) of changes** | **Details of changes made** |
| --- | --- | --- | --- | --- |
|  |  |  |  |  |

List details of all protocol amendments here whenever a new version of the protocol is produced.
