# Supplementary material for: Exploring the provision and structure of paediatric critical care outreach teams (PCCOTs) in the UK and Ireland: a national questionnaire study
Source: BMJ Paediatr Open. 2025 Dec 21;9(1):e003920. doi: 10.1136/bmjpo-2025-003920 (PMC12718590; doi:10.1136/bmjpo-2025-003920)
Supplement: online supplemental file 3 [file bmjpo-9-1-s003.docx]

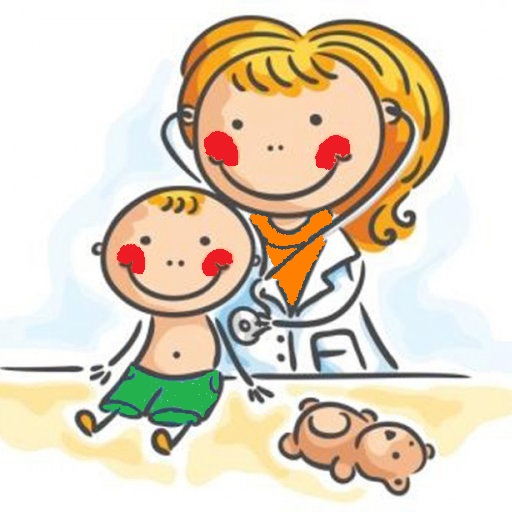
**DIVERTT** Study: **D**iscovery of **I**deal **V**ariables and **DIVERRT**

**E**xcellence markers in paediatric **R**apid **R**esponse **T**eams

Chief Investigator: Bethan Holmes

**Participant Information Sheet – Questionnaire**

We would like to invite you to take part in a questionnaire for the ‘DIVERTT Study’.

Before you decide, it is important for you to understand why we are doing the study and what will be involved if you decide to take part.

Please take time to read this information and discuss it with others if you wish. If there is anything that is not clear, or if you would like more information, please contact us via the contact details are at the end of this information sheet.

**What is the purpose of the DIVERRT study?**

The study focus is the paediatric Rapid Response Team (RRT) who are a group of professionals that are activated on the identification of a deteriorating patient. We would like to understand the geographic locations of the paediatric RRTs in the United Kingdom (UK) and Republic of Ireland (ROI), the characteristics of RRT professionals and how quality of care is measured within these teams. By participating in the study, you will assist in developing an understanding of where paediatric RRT are located, what the teams look like and how they are managed and utilised locally. This is the first research study to look at this area of practice.

The study is being undertaken by a paediatric nurse, Mrs Bethan Holmes, who works within the paediatric RRT at Birmingham Women’s and Children’s NHS Foundation Trust (BWCH). This work has been funded by the National Institute of Health Research and forms part of a training program called a Pre doctoral Clinical Academic Fellowship (PCAF). The project is being supervised by four experienced professionals: two senior clinicians with extensive research experience, Dr Heather Duncan and Dr Julie Menzies, who work at BWCH and two paediatric nurse academics with extensive clinical experience, Dr Susan Neilson and Dr Lucille Kelsall-Knight, who work at University of Birmingham.

**Why have I been invited to participate in the DIVERRT study?**

You have been invited to participate as you are a member of registered staff who works within a RRT in the UK and ROI . Your contact details were given to our team by either the Nurse in charge of your Paediatric Intensive Care Unit or your hospital’s Resuscitation Officer.

**Do I have to take part in the DIVERRT Study?**

No. Participation is entirely voluntary.

If you chose to participate, you could provide important information to aid in increasing understanding of paediatric RRT in the UK and ROI. This an area which has not previously been explored through research.

Please note, if you chose to take part then change your mind within seven days after participating, the information you provide will not be analysed. However, if you withdraw after the seven day period, the provided data may have been anonymised and analysis commenced, and data retrieval will not be possible.

**What will happen if I decide to take part in the DIVERRT Study?**

This is a two phase study and includes a questionnaire and interview.

The study is currently in phase one. We are asking for the completion of a short electronic questionnaire about the RRT you work within. This should take approximately 15 minutes to complete and can be accessed on a desktop or laptop computer or a smart phone.

Please note, the questionnaire will ask information about the following in your hospital :

- Number of hospital admissions per year,
- Number of inpatient beds,
- Number of referrals to the RRT per year,
- Common RRT referral reasons.

Please note, it would be useful to have gathered this information before starting the questionnaire as saving when partially through is not possible due to the software used.

Please note, it is possible to complete the questionnaire as a printed version and return to the research team by scanned document via email or via post. If you would prefer this option, please contact the research team on the details below and we would be happy to organise this for you.

At the end of the questionnaire, you will be asked if you would like to be considered for the second phase of the study, which is a semi-structured interview undertaken over video conferencing platform such as Zoom and will take 45-60 minutes. You will be asked to provide your email address and complete a short electronic consent form. Please note, the consent form just allows us to store your contact details. Within eight weeks of questionnaire completion, the research team will contact you with more information about the next phase of the research project prior to asking for further consent to be interviewed.

**What review process has the study undergone?**

The study has undergone four levels of review:

- Study design and questionnaire review by the local patient and public involvement (PPI) group called the Young Personal Advisory Group (YPAG) based at BWCH.
- Peer review of the questionnaire from local and national experts in quantitative research methods.
- The questionnaire has been piloted by the paediatric RTT at BWCH
- The study has received favourable review from University of Birmingham Ethics review.

**Will the information I give you be kept confidential?**

We are responsible for looking after your information and using it properly. The information you share is of great importance and is assured to be kept confidential. The information you share with us within the questionnaire will be analysed alongside other completed questionnaires and will result in descriptive outcome data such as percentages and counts. The researcher will anonymise the data you provide. Anonymised data will be stored on a password protected folder on the secure NHS server. Anonymised data will be shared with researcher’s supervisory team during the analysis stage. The study sponsor and regulatory bodies will access the data where relevant. Participants will not be identified on any shared data or future study dissemination.

If you have any concerns about this study please contact:

Please contact Mrs.Priyanka Batra:

Research Governance and Ethics Manager

Research Support Group

Room 119, Aston Webb Building

University of Birmingham

Edgbaston B15 2TT

Email: [researchgovernance@contacts.bham.ac.uk](https://mail.bham.ac.uk/owa/ADM-researchgov@adf.bham.ac.uk/redir.aspx?C=tjT9QF5vpA0ciulS5weEZVm-qOFDN1mfM-xEkM_o4bCIl5wwh6PUCA..&URL=mailto%3aresearchgovernance%40contacts.bham.ac.uk)

Phone: 0121 415 8011

Thank you for reading this information.

For further information, please use the following details to contact Bethan Holmes:

Email: bethan.holmes@nhs.net

Address: Bethan Holmes, Site Practitioner Team, Hospital Operation Centre, Birmingham Children’s Hospital, Steelhouse Lane, Birmingham. B4 6NH.

Phone: 0121 333 8018
