# Supplementary material for: Exploring the provision and structure of paediatric critical care outreach teams (PCCOTs) in the UK and Ireland: a national questionnaire study
Source: BMJ Paediatr Open. 2025 Dec 21;9(1):e003920. doi: 10.1136/bmjpo-2025-003920 (PMC12718590; doi:10.1136/bmjpo-2025-003920)
Supplement: Supplementary file 4 [file bmjpo-2025-003920.reviewer_comments.pdf]

PEER REVIEW HISTORY

BMJ Paediatrics Open publishes all reviews undertaken for accepted manuscripts. Reviewers are asked to complete a checklist review form and are provided with free text boxes to elaborate on their assessment. These free text comments are reproduced below.

ARTICLE DETAILS

**TITLE (PROVISIONAL)**	EXPLORING THE PROVISION AND STRUCTURE OF PAEDIATRIC CRITICAL CARE OUTREACH TEAMS (PCCOTS) IN THE UK AND IRELAND: A NATIONAL QUESTIONNAIRE STUDY
**AUTHORS**	Holmes, Bethan; Menzies, Julie; Neilson, Susan; Duncan, Heather; KELSALL-KNIGHT, LUCILLE

VERSION 1 - REVIEW

**REVIEWER NAME**	Peter Davis
**REVIEWER AFFILIATION**	Bristol Royal Hospital for Children, PICU
**REVIEWER CONFLICT OF INTEREST**	N/A
**AI DISCLOSURE**	No
**DATE REVIEW RETURNED**	18-Aug-2025

**GENERAL COMMENTS**	This study has attempted to better describe the situation with Paediatric Critical Care Outreach Teams (PCCOTs) across the UK, and found that they remain in a minority even in tertiary paediatric centres. The questionnaire has also exposed significant differences in the functioning and constitution of these teams, and it must remain a concern that only 7 out of 29 centres reported having a 24/7 service. It should also be noted that there is the potential for misunderstanding in the nomenclature between PCCOTs and Rapid Response Teams (RRTs) as the two may also function as different teams in the same institution as part of a staged response, the latter being more likely to include a medical presence as per the in-patient National Paediatric Early Warning System (NPEWS) escalations in England. The authors have also noted that the increasing number of children with chronic, complex conditions underlines the risks of delayed identification of deterioration and the importance of having this support available.<br>The authors have also mentioned the inconsistency in quality metrics and clinical governance relating to these teams, related to the challenges faced in demonstrating the benefit of these teams, and have rightly suggested that further work needs to be done to identify appropriate and objective measures of impact. Linking PCCOTs to a Critically Ill Child / Deteriorating Patient hospital-wide group recommended in the Paediatric Critical Care Get It Right FIrst Time (GIRFT) report of April 2022 referenced in the discussion might be one way to provide the desired level of clinical governance oversight.<br>In the time since this survey was undertaken there have also been significant changes to the approach to deteriorating paediatric patients, particularly in England, with the introduction of the in-patient NPEWS (as noted above) and more latterly Martha's Rule, both of which require some form of outreach support to children for review and escalation. It would seem sensible for the authors to mention this somewhere within their discussion. Likewise whilst

	developing a core metric dataset for further research may also be beneficial, the authors might also consider recommending that future editions of relevant professional standards (i.e. the Quality Standards for the Care of Critically Ill or Injured Children of the Paediatric Critical Care Society) include more details on the configuration of PCCOTs and their governance arrangements.

VERSION 1 – AUTHOR RESPONSE

Reviewers Feedback	Authors Response
There is the potential for misunderstanding in the nomenclature between PCCOTs and Rapid Response Teams (RRTs) as the two may also function as different teams in the same institution as part of a staged response, the latter being more likely to include a medical presence as per the in-patient National Paediatric Early Warning System (NPEWS) escalations in England.	We thank the reviewer for highlighting the potential for confusion between PCCOTs and RRTs. We agree these may function as distinct entities within a staged escalation pathway, with RRTs often including a medical presence. To avoid ambiguity, we have clarified this distinction in the manuscript and ensured consistent use of nomenclature.<br><br>In the Introduction Section, we now state;<br><br>**While these terms are sometimes used interchangeably, in some institutions they may represent distinct stages of escalation, with RRTs more likely to include a medical presence.**
The authors have also mentioned the inconsistency in quality metrics and clinical governance relating to these teams, related to the challenges faced in demonstrating the benefit of these teams, and have rightly suggested that further work needs to be done to identify appropriate and objective measures of impact. Linking PCCOTs to a Critically Ill Child / Deteriorating Patient hospital-wide group recommended in the Paediatric Critical Care Get It Right FIrst Time (GIRFT) report of April 2022 referenced in the discussion might be one way to provide the desired level of clinical governance oversight.	Thank you for your feedback and suggestions for further explain future steps to ensure clinical governance oversight of PCCOT and tying to current national recommendations.<br><br>Within the Discussion section, we now state;<br><br>**As an interim approach, linking PCCOTs to a hospital-wide Deteriorating Patient Working Group, as recommended by the Paediatric Critical Care GIRFT report[10], may strengthen clinical governance oversight**.<br><br>Within the Conclusion section, we have added;

	**Measures are required at both local and national levels: linking PCCOTs to hospital-wide Deteriorating Patient Groups can strengthen local governance,** while professional standards should provide clearer guidance on PCCOT configuration and oversight.
In the time since this survey was undertaken there have also been significant changes to the approach to deteriorating paediatric patients, particularly in England, with the introduction of the in-patient NPEWS (as noted above) and more latterly Martha's Rule, both of which require some form of outreach support to children for review and escalation. It would seem sensible for the authors to mention this somewhere within their discussion.	Thank you for this valuable feedback, which underscores the ongoing importance of PCCOT within the 2025 healthcare context.<br><br>Within the Discussion section, we now state;<br><br>**Since this study was conducted in 2022, management of deteriorating paediatric patients in England has evolved with the introduction of the inpatient National Paediatric Early Warning Score (NPEWS) and Martha's Rule[6], both requiring PCCOT support, reinforcing the relevance of the study's findings.**
Likewise whilst developing a core metric dataset for further research may also be beneficial, the authors might also consider recommending that future editions of relevant professional standards (i.e. the Quality Standards for the Care of<br><br>Critically Ill or Injured Children of the Paediatric Critical Care Society) include more details on the configuration of PCCOTs and their governance arrangements.	Thank you very much for this insightful comment.<br><br>Within the Discussion Section, we now state;<br><br>**Future editions of relevant professional standards, such as those from the PCCS and NOrF, should provide clearer guidance on PCCOT configuration and governance arrangements.**<br><br>Within the Conclusion section, we have added;<br><br>Measures are required at both local and national levels: linking PCCOTs to hospital-wide Deteriorating Patient Groups can strengthen local governance, **while professional standards**

	**should provide clearer guidance on PCCOT configuration and oversight.**

VERSION 2 - REVIEW

**REVIEWER NAME**	Peter Davis
**REVIEWER AFFILIATION**	Bristol Royal Hospital for Children, PICU
**REVIEWER CONFLICT OF INTEREST**	N/A
**AI DISCLOSURE**	No
**DATE REVIEW RETURNED**	29-Nov-2025

**GENERAL COMMENTS**	Thank you to the authors for taking on board the previous reviewer comments and included mention of both Martha's Rule and the National Paediatric Early Warning System in the discussion, so as to show the relevance of PCCOTs going forward.<br>There is one small typo in the table on Page 8, line 33: "unut" should read as "unit".
